# The development of functional mapping by three sex-related loci on the third whorl of different sex types of *Carica papaya* L.

**DOI:** 10.1371/journal.pone.0194605

**Published:** 2018-03-22

**Authors:** Chen-Yu Lee, Hui-Jun Lin, Kotapati Kasi Viswanath, Chih-Peng Lin, Bill Chia-Han Chang, Pei-Hsun Chiu, Chan-Tai Chiu, Ren-Huang Wang, Shih-Wen Chin, Fure-Chyi Chen

**Affiliations:** 1 Department of Plant Industry, National Pingtung University of Science and Technology, Pingtung, Taiwan; 2 Yourgene Bioscience, Shu-Lin District, New Taipei City, Taiwan; 3 Pingtung Seed & Seedling Research Center, Taiwan Seed Improvement and Propagation Station, Pingtung, Taiwan; 4 Kaohsiung District Agricultural Research and Extension Station, Council of Agriculture, Pingtung, Taiwan; University of Naples Federico II, ITALY

## Abstract

*Carica papaya* L. is an important economic crop worldwide and is used as a model plant for sex-determination research. To study the different flower sex types, we screened sex-related genes using alternative splicing sequences (AS-seqs) from a transcriptome database of the three flower sex types, i.e., males, females, and hermaphrodites, established at 28 days before flowering using 15 bacterial artificial chromosomes (BACs) of *C*. *papaya* L. After screening, the cDNA regions of the three sex-related loci, including short vegetative phase-like (*CpSVPL*), the chromatin assembly factor 1 subunit A-like (*CpCAF1AL*), and the somatic embryogenesis receptor kinase (*CpSERK*), which contained eight sex-related single-nucleotide polymorphisms (SNPs) from the different sex types of *C*. *papaya* L., were genotyped using high-resolution melting (HRM). The three loci were examined regarding the profiles of the third whorl, as described below. *CpSVPL*, which had one SNP associated with the three sex genotypes, was highly expressed in the male and female sterile flowers (abnormal hermaphrodite flowers) that lacked the fourth whorl structure. *CpCAF1AL*, which had three SNPs associated with the male genotype, was highly expressed in male and normal hermaphrodite flowers, and had no AS-seqs, whereas it exhibited low expression and an AS-seqs in intron 11 in abnormal hermaphrodite flowers. Conversely, carpellate flowers (abnormal hermaphrodite flowers) showed low expression of *CpSVPL* and AS-seqs in introns 5, 6, and 7 of *CpSERK*, which contained four SNPs associated with the female genotype. Specifically, the *CpSERK* and *CpCAF1AL* loci exhibited no AS-seq expression in the third whorl of the male and normal hermaphrodite flowers, respectively, and variance in the AS-seq expression of all other types of flowers. Functional mapping of the third whorl of normal hermaphrodites indicated no AS-seq expression in *CpSERK*, low *CpSVPL* expression, and, for *CpCAF1A*L, high expression and no AS-seq expression on XY^h^-type chromosomes.

## Introduction

Papaya (*Carica papaya* L., 2*n* = 18) belongs to the family Caricaceae, which includes 35 species within six genera and is an extensively cultivated fruit in tropical and subtropical zones. It is a polygamous flowering plant with a small genome of 372 Mbp [1]. Papaya trees grow fruit throughout the year, which has high nutritional benefits and numerous medicinal applications. It is a trioecious plant with male, hermaphrodite, and female sex forms. In many regions of the world, cultivation of hermaphrodite papaya has several advantages versus female or male papaya, including lower cost and higher yield, as all trees will produce fruit. However, hermaphrodite papaya is unstably transformed, as female sterile flowers and carpellate flowers form deformed carpellodic fruits because of the effect of environmental factors, such as temperature and soil fertility [2]. The development of stable hermaphrodite cultivars is crucial for papaya breeding.

Papaya is an outstanding model system to study sex determination because of its excellent characteristic features, including a comparatively short life-cycle, small genome size, and readily available genetic information. According to the genetic hypothesis of sex determination proposed independently by Storey and Hofmeyr, the sex type of papaya is controlled by a single locus with at least three alleles: *M*_*1*_, a dominant allele for male plants; *M*_*2*_, a different dominant allele for hermaphrodite plants; and *m*, a recessive allele for female plants. All combinations of dominant alleles, i.e., *MM*, *MM*^*h*^, and *M*^*h*^*M*^*h*^ (where *M* represents the male and *M*^*h*^ represents the hermaphrodite alleles) are lethal to the zygote [3–5].

High-resolution melting (HRM), a novel post-PCR technique, has been recently implemented in molecular marker technology. HRM has several advantages over other genotyping technologies: it is simpler, flexible, nondestructive, highly sensitive, specific, and cost-effective [6]. A recent enhancement in HRM allows the detection of differences in single nucleotides and the performance of high-throughput mutation scanning and genotyping by characterizing sensitive melting curves at high resolution [6]. Based on the convenience of HRM, genetic polymorphism in various plant species has been analyzed. It has been used to detect single sequence repeats (SSRs) in grapevine, and SSRs and SNPs in olive cultivars and citrus plants [7]; to analyze genetic polymorphism in peach species [8]; to identify specific genes in the breeding of tomato (*Solanum lycopersicum*) mutants [9]; to genotype 25 almond cultivars with EST-SNPs, InDels, and SSRs [7]; to draw the genetic linkage maps of white lupin (*Lupinus albus*) [10]; to identify different cultivars of *Artemisia* species [11]; and to identify outcrossing tetraploid genotypes of alfalfa [12].

Recent developments in next-generation sequencing (NGS) and the analysis of massive sequence information, small non-coding RNAs (sRNAs), and the transcriptome have effectively improved the diagnostics of numerous genes involved in sex determination in *C*. *papaya* L. [13–15]. For instance, Urasaki *et al*. (2012) identified the *Cp2671* gene based on transcriptome assembly in *C*. *papaya* L. floral organs using Ht-SuperSAGE in combination with SOLiD sequencing technology [15]. The encoded Cp2671 protein shows high similarity (85%) with the short vegetative phase (SVP) protein of *Arabidopsis thaliana*, as this gene contains a conserved MADS-box family domain and is in the male-specific region of the Y chromosome (MSY).

In the present study, two genes that are believed to be sex-related genes were identified according to the NGS results: the *SVP* (*CpSVPL*) and somatic embryogenesis receptor kinase (*CpSERK*) genes. *CpSVPL* is a candidate gene for sex determination in *C*. *papaya* L. [15]. In *A*. *thaliana* and other flowering plants, the *SVP* gene plays an important role in the response of plants to ambient temperatures with respect to regulating the flowering time and affects the development of the B and C models. *SVP* also controls the transition of plants from vegetative growth to reproductive growth [16–18]. SERKs are a large family of highly conserved, leucine-rich repeat receptor-like kinases (LRR-LKs). To date, various SERK genes that play significant roles in plant growth and development have been identified. Among these roles of SERKs, male sporogenesis, separation of floral organs, and embryo development are important for plant reproduction [19–22]. The intergenic distance between the *SVP* and *SERK* genes is approximately 26000 bp (S1 Fig).

The sex of papaya plants is genetically controlled by a sex-specific chromosome region (X, Y^m^, and Y^h^) that behaves like an XX, XY^m^, and XY^h^ chromosome with respect to female, male, and hermaphrodite flower development, respectively [23, 24]. Y-chromosomal gene families are expressed specifically in the testes in humans [25]. The Y chromosomes of fruit flies (*Drosophila melanogaster*) occur as a result of Y-linked regulatory variation [26] and the transcriptional inactivation of X chromosomes is associated with the chromatin patterns of Y chromosomes [27–29]. With respect to transcription levels, the chromatin assembly factor 1 (CAF-1) complex is conserved in plants, and its subunit A (CAF1A) regulates DNA methylation and RNA alternative splicing (AS) [30, 31]. A link between alternative splicing sequences (AS-seqs) and protein diversity has been suggested, which could affect plant phenotypes further. Any impairment in the CAF-1 complex causes multiple phenotypes in plants, as shown for both *A*. *thaliana* [32] and rice [33]. Kwon *et al*. (2014) demonstrated that AS is a molecular scheme that is governed by environmental factors, such as temperature, light, and soil minerals [34].

The polygamous nature of papaya offers various advantages for genetic and evolutionary studies, as well as for sex determination, which is directly associated with efficient commercial fruit production. Our strategy involved the use of the transcriptome database of flower buds of the three sex types to screen genes that exhibited expression of the three sex-related AS-seqs in *C*. *papaya* L. The selected genes could be used to develop a functional map of the characteristics of the flowers of different sex types via genotyping and expression profiling. Furthermore, the results of functional mapping could be applied as a potential tool for marker-assisted selection of the stable hermaphrodite flowers in *C*. *papaya* L.

## Materials and methods

### Plant material and sample preparation

In this study, papaya flowers at 28 and 0 days before flowering were collected from the Pingtung Seed and Seeding Research Center (Pingtung, Taiwan). The five sex-type samples collected 28 days before flowering with flowers with a size of 0.2 cm included female (F), male (M), hermaphrodite (H), female sterile (HM), and carpellate (HF) flower types. The seven flower samples (F4, M3, H4, H3, HM3, HF4, and HF3) collected 0 days before flowering were divided into third and fourth whorl tissues.

### mRNA extraction and cDNA synthesis

From the 12 samples described above, mRNA was isolated and treated with RNase-free DNase I to eliminate contaminating DNA. The concentration and quality of the isolated mRNA samples were measured by 1% agarose gel electrophoresis and UV digital imaging (Digigel; Shan Shui Technology Ltd., Tainan, Taiwan). After quantification and quality checking, the 12 mRNA samples were subjected to a reverse transcription reaction by adding random primers (Protech, Taipei, Taiwan) and RTase, to amplify the target cDNA using a Labcycler thermocycler (SensoQuest, Göttingen, Germany). The conditions used for reverse transcription were as follows: 10 min at 70°C, 3 min at 4°C, 3 min at 42°C, 10 min at 25°C, 90 min at 42°C, and 5 min at 85°C.

### Transcriptome sequence assembly and annotation

The mRNAs extracted from the three sex types (males, females, and hermaphrodites) of the flower buds collected 28 days before flowering exhibited a purity ranging between 1.9 to 2.1 and an RNA integrity number (RIN) >8.0 and were sent to Yourgene Bioscience on dry ice (New Taipei City, Taiwan), for cDNA synthesis. The synthesized cDNA samples were subjected to transcriptome sequencing, and a cDNA library was constructed using NGS technology on the Illumina HiSeq 2000 platform. Assembly of sequences and gene annotation were performed at Yourgene Bioscience. All data were output as Excel dataset files (S1 Dataset). The transcriptome sequence data were submitted to GenBank. The accession numbers for the transcriptome sequence data are: ap1 SRR6416941 (hermaphrodite), ap2 SRR6416942 (female), ap3 SRR6416943 (male), ap4 SRR6416944 (hermaphrodite), and ap5 SRR6416945 (male).

### Gene functional mapping from the transcriptomes of the flowers of three sex types

We searched the BAC database of *C*. *papaya* L. on the NCBI website and screened the BACs that contained the genes without expression in females but with expression in the other sex types from our transcriptome data. We selected 15 BACs of *C*. *papaya* L. based on different expression levels, including the Y^h^ chromosome BAC-49L11, X chromosome BAC-50J21, Y^h^ chromosome BAC-50M09, Y^h^ chromosome BAC-53G04, Y chromosome BAC-57M14, Y^h^ chromosome BAC-62H24, Y^h^ chromosome BAC-65D15, Y^h^ chromosome BAC-71E16, Y^h^ chromosome BAC-72J22, Y^h^ chromosome BAC-81O12, Y^h^ chromosome BAC-PH85B24, Y^h^ chromosome BAC-90D06, Y chromosome BAC-PH94E22, Y^h^ chromosome BAC-PH95B12, and Y^h^ chromosome BAC-96A24. Each BAC was used in the genetic linkage mapping of the three sex types from the transcriptome data using MapDraw (MS Excel; Microsoft, Redmond, WA).

### Screening of sex-related genes and sequence structure analysis

We used MS Excel to identify the alleles that expressed different sequences in BACs of different sex types, and to run the bioinformatics program ClustalW2 (http://www.ebi.ac.uk/Tools/msa/clustalw2/). Three types were identified according to the sequences of the expressed genes: 1) genes that were expressed in two sex types and encoded different amino acid sequences (AA-seqs) in the two sex types (type_1); 2) genes that were expressed in all sex types and encoded different AA-seqs in them (type_2); and 3) genes that were expressed in all sex types and encoded the same AA-seqs in any two of the three sex types (type_3). Candidate genes were selected based on the expression of the SNPs in type_1, type_2, and type_3. Subsequently, we screened for sex-related genes based on a literature search of the candidate genes. In addition, we predicted the functional domains of the proteins encoded by these screened genes to complete the structural map of the different sex types using the bioinformatics program SMART (http://smart.embl-heidelberg.de/).

### Primer design

Primers were designed using Beacon Designer™. Eight and two pairs of primers were designed for sex-related SNPs for the SNP-HRM and PCR–restriction fragment length polymorphism (PCR–RFLP) assays, respectively (S4 Table). The cDNA sequences of the three sex-related genes were designed for every junction as a forward primer paired with a reverse primer (S5 Table) for real-time PCR assays (qRT–PCR). Subsequently, the sequences were used as a forward primer on junction 1 and as a reverse primer for junction 2 for intron 1 tests of RT–PCR assays.

### SNP-HRM and genetic analyses

We analyzed the sex-related SNPs using genomic data (data not shown). In addition, we performed an SNP-HRM analysis in 12 samples with identified sex types (including parents and progenies). The reaction mixture in each tube included 10 μL of Precision Melt Supermix (Bio-Rad), 0.4 μL of 10 μM forward primer, 0.4 μL of 10 μM reverse primer, 3 μL (1.5 ng/μL) of DNA template, and ddH_2_O up to a total volume of 15 μL. The tubes were placed in a 96-well plate (BIOplastics) and sealed with a sealing membrane (BIOplastics). The RT–PCR conditions were as follows: 95°C for 2 min, followed by 28 cycles at 95°C for 10 s (for denaturation) and 57°C for 30 s (for annealing and elongation). The PCR conditions used to obtain melting curves were as follows: 95°C for 30 s, followed by 60°C for 1 min (for denaturation) and a temperature increment from 65°C to 90°C at the rate of 0.2°C/10 s, for the fluorescence detection of DNA double strands. The results were subjected to a cluster analysis using the Bio-Rad Precision Melt Analysis software to calculate correlations (%) among the three sex types. We then selected the SNP-HRM results of samples with a correlation of 100%. In addition, we selected 96 samples from the hybrid progenies of a hermaphrodite and a male parent for blind experiments. We calculated the number of the three sex types according to the results of the SNP-HRM analysis, followed by a chi-squared test to determine whether the segregation ratios of the sex characters tagged by SNP-HRM matched the theoretical values.

### PCR–RFLP analysis

The primers used for PCR–RFLP analysis were designed based on the sequences of the SNPs, and the product sizes were approximately 200 bp. Each tube comprised the following PCR constituents: 5 μL of 10× PC2 buffer (500 mM Tris-HCl [pH = 9.1], 160 mM (NH_4_)_2_SO_4_, 35 mM MgCl_2_, and 1.5 mg/mL bovine serum albumin [BSA]), 1 μL of 10 mM dNTP, 1 μL of 10 mM forward primer, 1 μL of 10 mM reverse primer, 1 μL of Taq DNA polymerase (2 U/μL), 2 μL of DNA template, 5 μL of dimethyl sulfoxide (DMSO), and ddH_2_O up to a total volume of 50 μL. The PCR reaction mixture was pipetted into 0.2 mL microfuge tubes that were transferred to a PCR machine (BIOER-GenePro). The conditions of PCR–RFLP were as follows: 94°C for 3 min (for initial denaturation), followed by 35 cycles at 94°C for 40 s (for denaturation), 61°C for 40 s (for annealing), and 72°C for 30 s (for elongation). The amplified PCR products were purified using a purification kit (Geneaid). We then examined the concentration of the PCR products. Four hundred nanograms of PCR product was reacted with restriction enzymes for the analyses. The PCR products of *CpSVPL* and *CpSERK* genes were digested with the Nde I and Spe I enzymes, respectively, using a suitable buffer at 37°C/8 h. Individual electrophoretic analyses of each state of the PCR product before and after the enzymatic reactions were performed, and UV images were acquired.

### PCR assays

We performed PCR assays to test the intron predictions for the 12 cDNA samples. The forward primer was designed for the sequences between two exons, and the reverse primer was designed for the sequences of the next exon. The PCR reaction mixture in each tube included the following constituents: 2.5 μL of 10× Taq buffer, 0.5 μL of 10 mM dNTP, 0.5 μL of 10 mM forward primer, 0.5 μL of 10 mM reverse primer, 0.5 μL of Taq DNA polymerase (2 U/μL), and 3 μL of cDNA template (80 ng/μL). The total volume of the reaction mixture was made up to 25 μL with ddH_2_O and the reaction mixture was pipetted into microfuge tubes, which were transferred to a PCR machine (SensoQuest Labcycler 96). The PCR conditions were as follows: 95°C for 4 min, 35 cycles of 94°C for 40 s, 58°C for 40 s, 72°C for 20–120 s (for elongation, depending on the length of the fragment), a final extension at 72°C for 3 min, and a hold at 4°C. Five microliters of the RT–PCR product from each tube was used for individual electrophoretic analyses, and UV images were taken.

### qRT–PCR assays

A qRT–PCR analysis was performed for each gene junction in the 12 cDNA samples (S7–S9 Tables). The qPCR mixture of each tube comprised the following constituents: 10 μL of master mix (Basic Life Science), 0.4 μL of 10 mM forward primer, 0.4 μL of 10 mM reverse primer, and 4 μL of cDNA sample (10 ng/μL). The total volume of the reaction mixture was made up to 20 μL with ddH_2_O. The mixtures were transferred to 96-well plates, which were then sealed with a sealing membrane. The plates were transferred to a qPCR machine (Bio-Rad CFX Connect) that was operated using a qPCR software (Bio-Rad CFX Manager). The qRT–PCR conditions were as follows: 95°C for 10 min, followed by 39 cycles of 95°C for 15 s (for denaturation) and 60°C for 1 min (for annealing and elongation). The conditions for the melting curve analysis were 95°C for 15 s, followed by 60°C for 30 s (for denaturation). The detection range was set from 65°C to 95°C, and the incremental speed was set to 0.5°C/10 s. Each tube of qRT–PCR product was subjected to individual electrophoretic analysis and UV images were taken.

## Results

### Screening of sex-related genes

**Fig 1:**
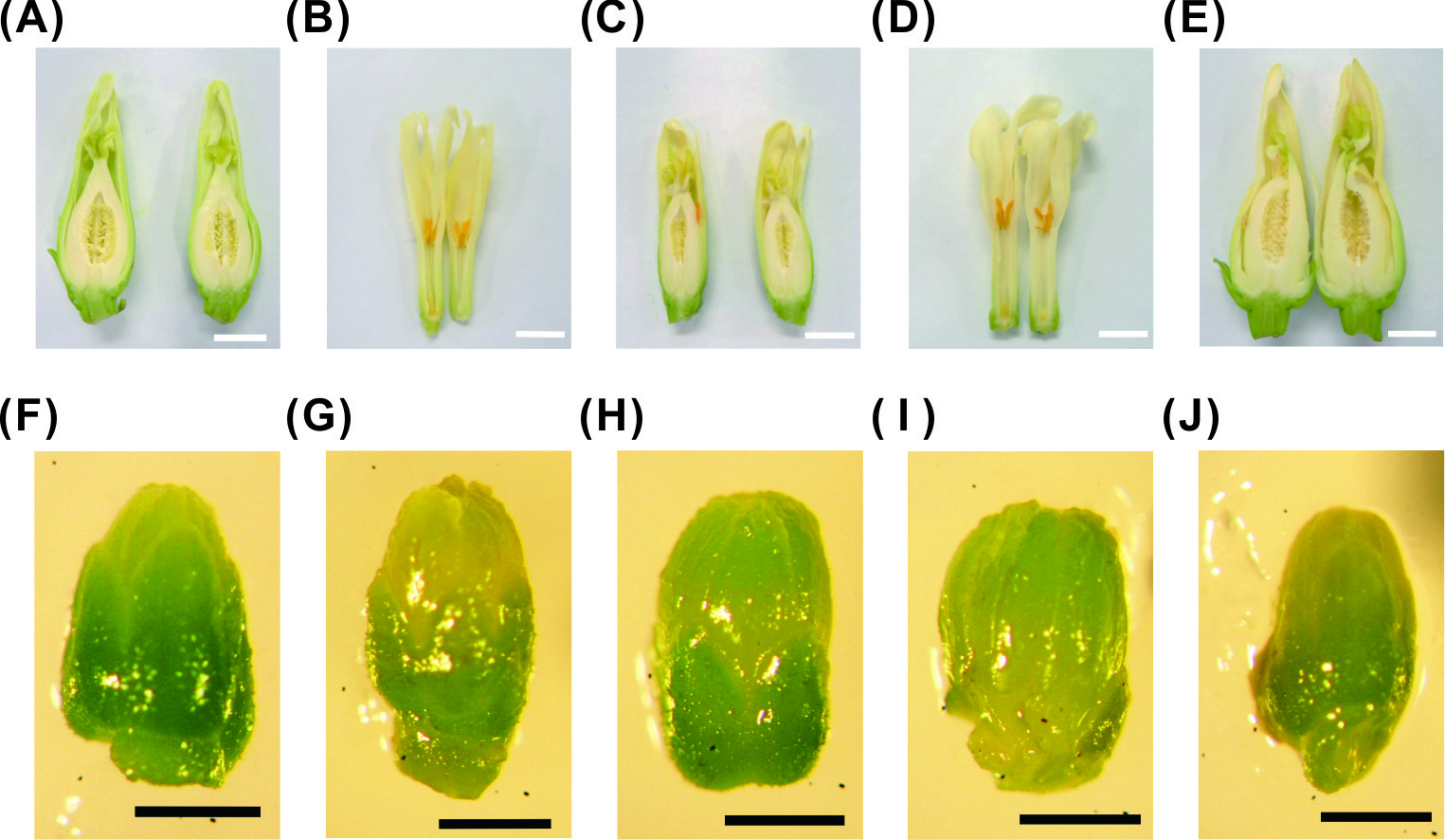
Flowers of the different sex types of papaya. (A) female; (B) male; (C) normal hermaphrodites; (D) carpellody hermaphrodite; (E) female degradation hermaphrodite; (F) 0.2 cm female buds; (G) 0.2 cm male buds; (H) 0.2 cm normal hermaphrodite buds; (I) 0.2 cm carpellody hermaphrodite buds; (J) 0.2 cm female degradation hermaphrodite buds.

**Fig 2:**
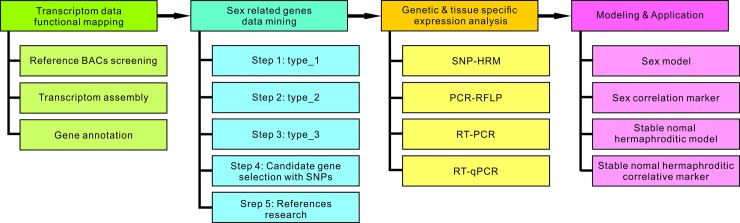
Experimental flowchart.

**Fig 3:**
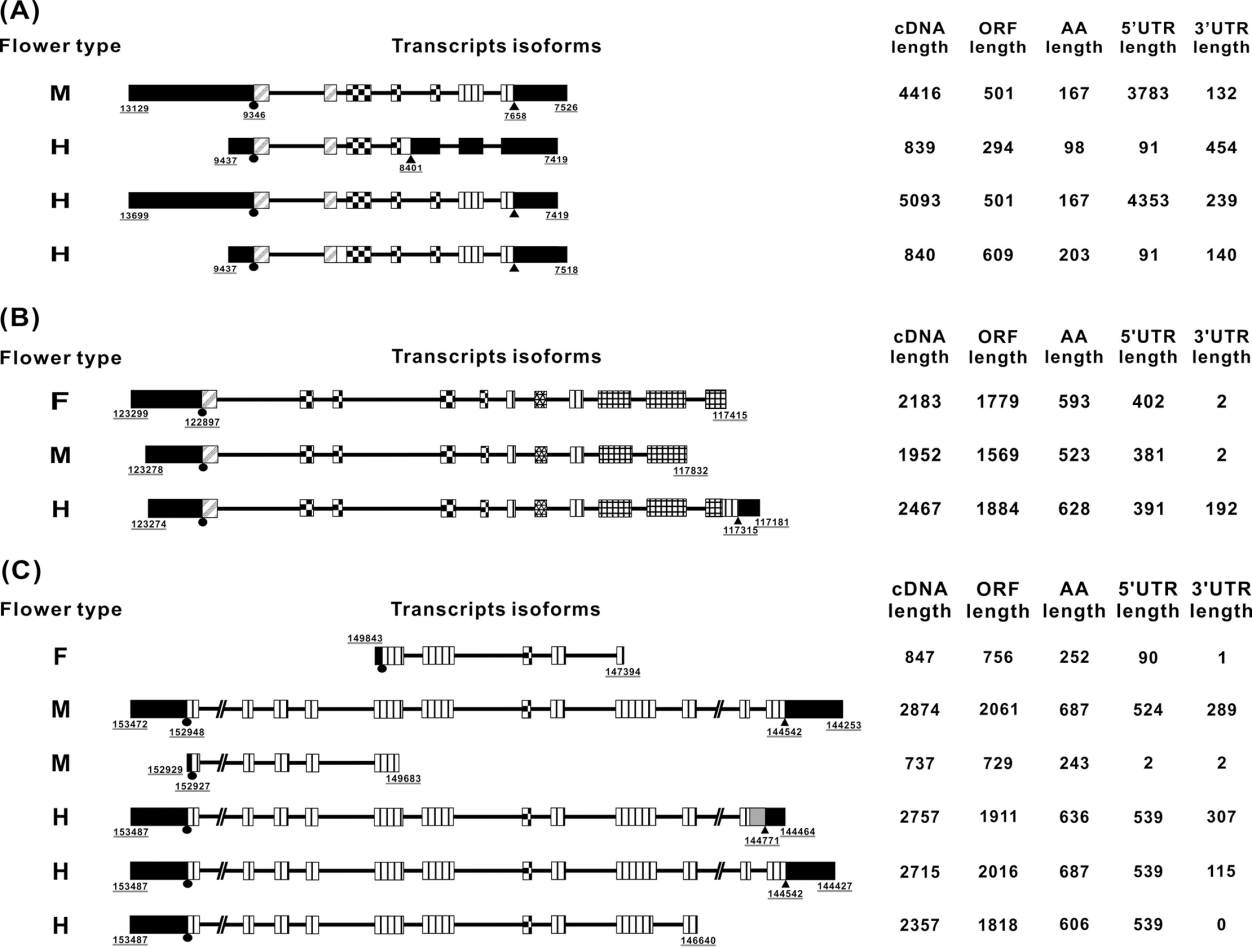
AS-seqs of the *CpSVPL*, *CpSERK*, and *CpCAF1AL* genes from the transcriptome sequences of the three sex types retrieved from the BACs of papaya from the national center for biotechnology information (NCBI). The cDNA region of the functional domain of the (A) *CpSVPL* gene is located on the Y^h^ chromosome of BAC 71E16. Square with diagonal strips: MADS-BOX regions; square with checkered pattern: K-BOX regions; (B) the *CpSERK* gene is located on the Y^h^ chromosome of BAC 71E16. Square with diagonal strips: signal peptide (SP) regions; square with checkered pattern: leucine-rich repeat (LRR) regions; oblique grid pattern: transmembrane regions (TMs); grid pattern: kinase regions (serine/threonine protein kinases); (C) the *CpCAF1AL* gene is located on the Y^h^ chromosome of BAC 50M09: square with diagonal strips: CAF-1 regions. The underlined numbers indicate the locations on the BACs. F: females; M: males; H: hermaphrodites; circles: start codons; triangles: stop codons; squares: exons; horizontal lines: introns; black squares: UTRs; squares with vertical stripes: non-specific regions. The nucleotide numbers and AA numbers are indicated on the right side of the structures. ORF: open reading frame; AA: amino acid; UTR: untranslated region. The underlined numbers indicate the nucleotide numbers.

**Fig 4:**
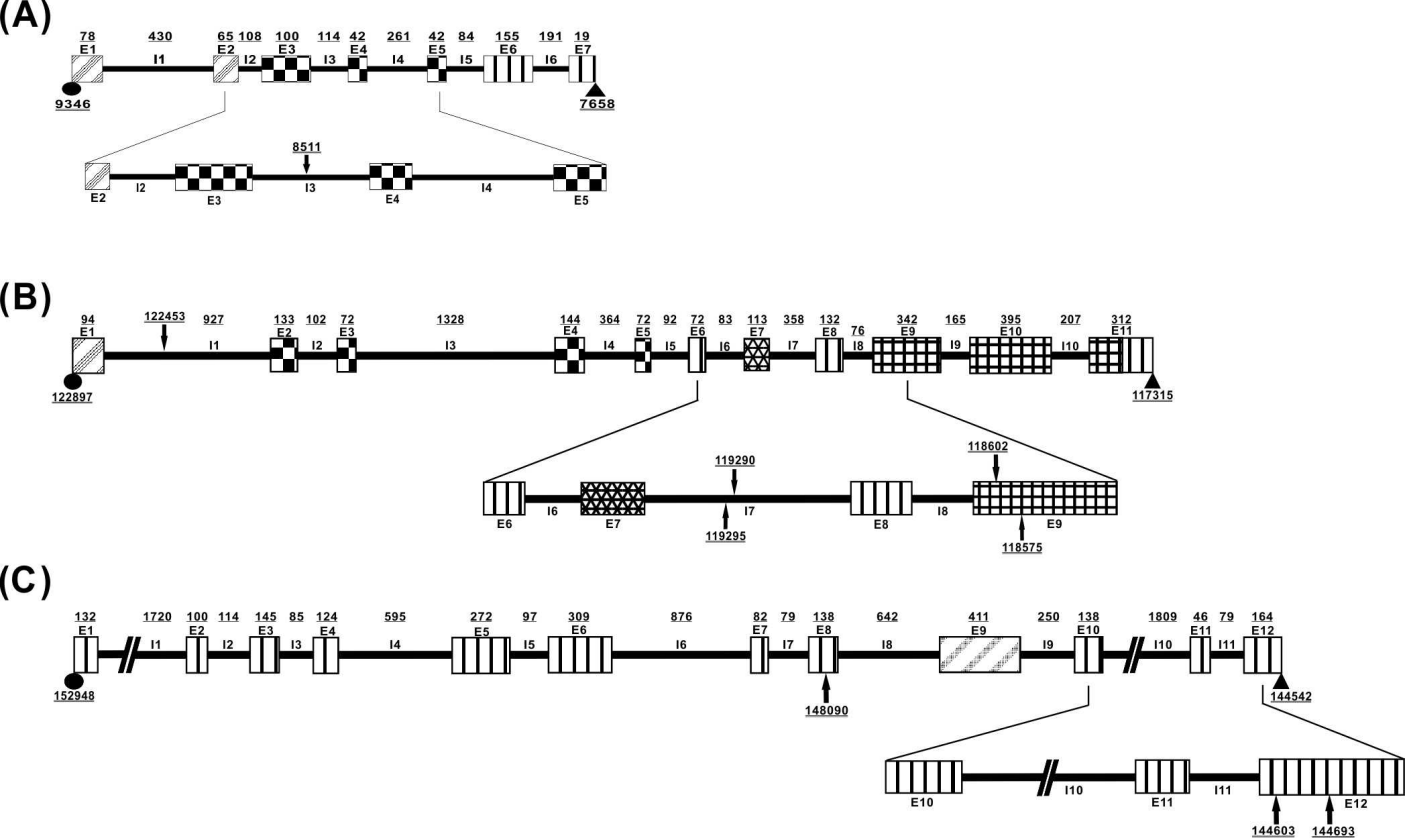
Position of sex-related SNPs within the cDNA regions of the sex-related genes of papaya. Coding region of the (A) *CpSVPL* gene on the Y^h^ chromosome of BAC 71E16; (B) of the *CpSERK* gene on the Y^h^ chromosome of BAC 71E16; and (C) of the *CpCAF1AL* gene on the Y^h^ chromosome of BAC 50M09. Arrows: SNP position; underlined numbers: locations on BACs; circles: start codons; triangles: stop codons; thick lines: locations of gene exons; thin lines: locations of gene introns; solid-black filled-in thick lines: UTRs; vertical-striped filled-in thick lines: non-specific regions; diagonal-striped filled-in thick lines (A): MADS-BOX regions; checkered-pattern filled-in thick lines (A): K-BOX regions; diagonal stripes in thick lines (B): SP; checkered pattern in thick lines (B): LRR; diagonal netting patterns in thick lines (B): TM; net patterns in thick lines (B): serine/threonine protein kinases and the kinase regions; diagonal stripes in thick lines (C): CAF-1 domain.

Flower organs were collected from the three sex types of *C*. *papaya* L. plants (Fig 1). The sex-related genes were screened based on 1) their differences in expression and sequence and 2) the genomic database of the three sex types (Fig 2). First, we completed the linkage map of the expressed genes according to the sequences of the transcriptome developed from the different sex types of young flower buds that were collected 28 days before flowering into the 15 BAC templates of *C*. *papaya* L. (Fig 3 and S1 Dataset). Among the 15 reference BACs, 117 genes were shown to be expressed in the three sex types (S1 Fig and S1 Dataset) and 55 genes were expressed in two and three sex types. The sequences of the 55 sex-related genes from the database (S1 Dataset) were analyzed using ClustalW2, which sorted the genes into four categories: exon jump, intron jump, AA short, and no AA-seq change; these categories contained 16, 8, 14, and 24 genes, respectively (S2 Table). The three types of AA-seq changes identified among the three sex types were classified according to their differences in expression: genes that were expressed in two sex types and had different AA-seqs between them (type_1), genes that were expressed in all three sex types but had different AA-seqs among them (type_2), and genes that were expressed in all three sex types and had the same AA-seqs between two of the three sex types (type_3). The number of genes of type_1, type_2, and type_3 were 13, 7, and 11, respectively (S3 Table). The final results of the sex-related loci, which involved the *CpSVPL*, *CpSERK*, and *CpCAF1AL* genes, indicated polymorphisms in AA-seqs in the flowers of the three sex types (Fig 3, S2, S3 and S4 Figs), and the NGS genotype analysis revealed the presence of sex-related SNPs (data not shown) in the coding region of these three sex-related genes (Fig 4 and Table 1).

**Table 1:** Sex correlation percentage and chi-squared analysis results.

SNP related sex type	BAC name	SNP position	Correlation (%)			Blind sample HRM test				
			F	H	M	F	H	M	Sex ratio	Square
3 sex type	Y^h^ chromosome BAC-71E16	8487	100%	100%	100%	31	31	30	1:1:1	0.01
Female type	Y^h^ chromosome BAC-71E16	122536	100%	94%	93%	42	54 (M + H)		1:2	4.69*
	Y^h^ chromosome BAC-71E16	119737, 119742	100%	100%	71%	26	54 (M + H)		1:2	0.02
	Y^h^ chromosome BAC-71E16	119048	100%	100%	67%	38	58 (M + H)		1:2	1.69
	Y^h^ chromosome BAC-71E16	119022	100%	100%	47%	35	61 (M + H)		1:2	0.42
Male type	Y^h^ chromosome BAC-50M09	144693	83%	83%	100%	76 (F + H)		35	2:1	0.16
	Y^h^ chromosome BAC-50M09	144403	83%	86%	100%	60 (F + H)		26	2:1	4.08*
	Y^h^ chromosome BAC-50M09	144403	100%	86%	100%	68 (F + H)		40	2:1	0.67

The progenies of male and hermaphrodite parental crosses from the SNP–HRM assay of the three sex types of papaya.

* Significance at *P* < 0.05.

### SNP correlative analysis of the three sex types

Regarding the results of the melting curves based on the SNP-HRM analysis, the primers (S4 Table) for the sex-related SNPs in the three sex-related loci could be used to identify the plants of all three sex types via the CpSVPL_HRM_88 primer at the *CpSVPL* locus; the female type via the CpSERK_HRM_34072, CpSERK_HRM_30704, CpSERK_HRM_34760, and CpSERK_HRM_34787 primers at the *CpSERK* locus; and the male type via the CpCAF1AL_HRM_01, CpCAF1AL_HRM_02, and CpCAF1A*L*_HRM_04 primers at the *CpCAF1AL* locus (S5 Fig). The results of both the genetic analysis of sex correlation and chi-squared tests revealed the identity of the three sex types, the female type, and the male type via the primers located at the *CpSVPL*, *CpSERK*, and *CpCAF1AL* loci, respectively, from the parents and progenies identified in the SNP-HRM analysis (Table 1 and S6 Fig). Regarding the results of the PCR–RFLP analysis, the three sex types were recognized using the *CpSVPL*_RFLP_Nde I and *CpSERK*_RFLP_Spe I primers (S4 Table; S4 and S7 Figs). No PCR products from the female plant were observed among the results of the PCR–RFLP analysis performed using the *CpSVPL*_RFLP_Nde I primer (data not shown).

### Expression analysis of the sex-related genes

**Fig 5:**
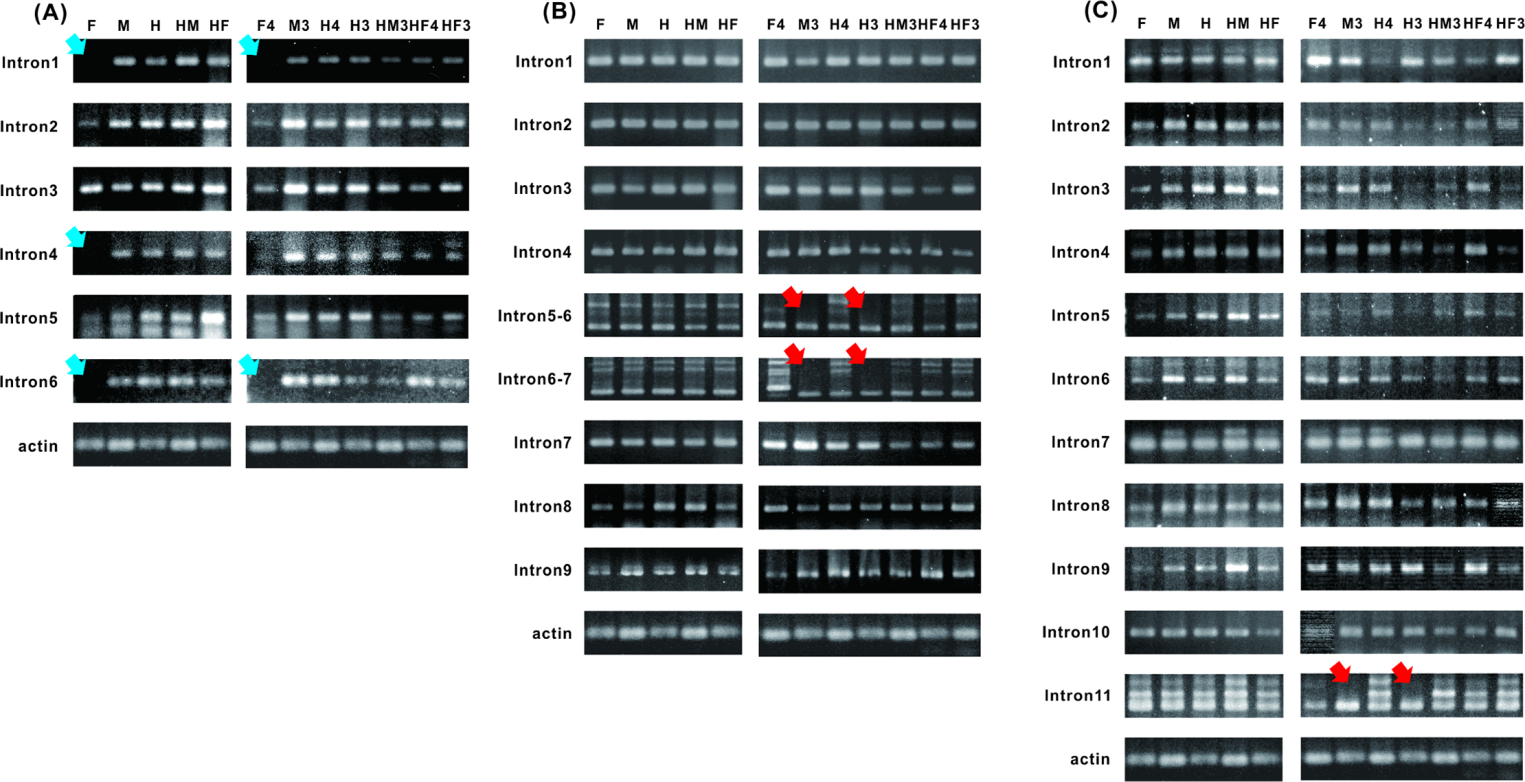
Results of the RT–PCR assay of the *CpSVPL*, *CpSERK*, and *CpCAF1AL* genes. (A) *CpSVPL* gene; (B) *CpSERK* gene; (C) *CpCAF1AL* gene. F: female buds at 28 days before flowering; M: male buds at 28 days before flowering; H: normal hermaphrodite buds at 28 days before flowering; HM: female degradation hermaphrodite buds at 28 days before flowering; HF: carpellody hermaphrodite buds at 28 days before flowering; F4: female fourth whorl; M3: male third whorl; H4: normal hermaphrodite fourth whorl; H3: normal hermaphrodite third whorl; HM3: female degradation hermaphrodite third whorl; HF4: carpellody hermaphrodite fourth whorl; HF3: carpellody hermaphrodite third whorl. The actin gene was used as a housekeeping control. Blue arrows indicate the absence of PCR products of no AS-seq cDNA; red arrows indicate the absence of PCR products of AS-seq cDNA.

**Fig 6:**
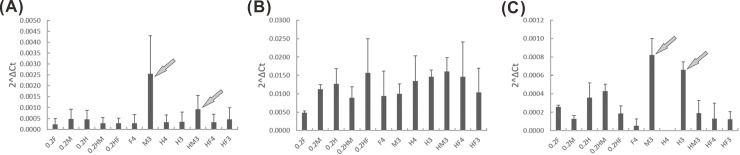
Results of the qPCR assay of the *CpSVPL*, *CpSERK*, and *CpCAF1AL* genes from different sex types. (A) Junction 6 of *CpSVPL*; (B) junction 10 of *CpSERK*; (C) junction 4 of *CpCAF1AL* in the qPCR assay. Arrows: significantly different expression; 0.2F: female buds at 28 days before flowering; 0.2M: male buds at 28 days before flowering; 0.2H: normal hermaphrodite buds at 28 days before flowering; 0.2HM: female degradation hermaphrodite buds at 28 days before flowering; 0.2HF: carpellody hermaphrodite buds at 28 days before flowering; F4: female fourth whorl; M3: male third whorl; H4: normal hermaphrodite fourth whorl; H3: normal hermaphrodite third whorl; HM3: female degradation hermaphrodite third whorl; HF4: carpellody hermaphrodite fourth whorl; HF3: carpellody hermaphrodite third whorl.

**Fig 7:**
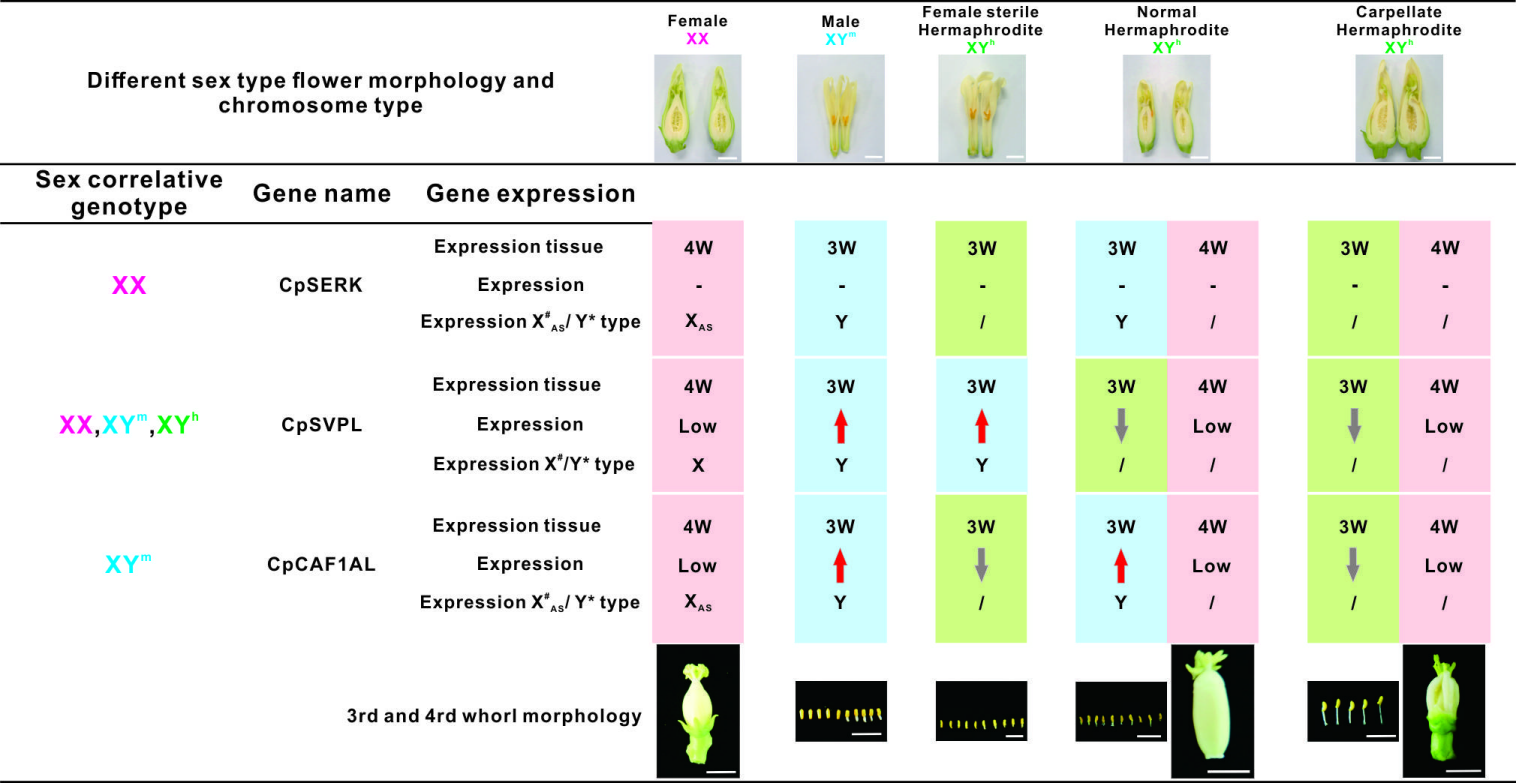
Sex-related gene expression in the third and fourth whorls of *C*. *papaya* L. flowers of different sex types. X: X chromosome; Y^m^: Y^m^ chromosome; Y^h^: Y^h^ chromosome; 4W: fourth whorl; 3W: third whorl; low: low expression in the same tissue;–: no difference in expression between the third and fourth whorls; *: no X-chromosome-specific expression at the locus; #: X-chromosome-specific expression at the locus; /: X- or Y-chromosome-specific expression at the locus; AS: AS-seq expression; red arrow: higher expression in the same tissue; gray arrow: lower expression in the same tissue; pink background: female expression type; blue background: male expression type; green background: hermaphrodite expression type.

We performed AS-seq and gene expression analyses using RT–PCR and qPCR, respectively, for the three sex-related genes. The sequences of the primers used in these analyses are shown in S8 Fig and S5 Table. The results indicated that only the tests of introns 3 and 5 of the *CpSVPL* gene in the fourth whorl of female flowers could be used to detect correctly the lengths of the fragments. Tests of introns 1 to 6 of the *CpSVPL* gene could be used to detect correctly the lengths of the fragments in the other sex types and abnormal hermaphrodite flowers, and there was no occurrence of AS. Therefore, we could distinguish female flowers from other-sex-type flowers based on this difference. Furthermore, the expression level of the *CpSVPL* gene was clearly elevated in the third whorl of both male and female sterile flowers. Thus, we could distinguish male and female sterile flowers from other-sex-type flowers based on this difference. There were occurrences of AS-seq polymorphism in introns 5, 6, 7, and 11 of the *CpSERK* and *CpCAF1AL* genes. In addition, polymorphism occurred in the fourth whorl of female and hermaphrodite flowers and the third whorl of abnormal hermaphrodite flowers. The expression level of the *CpCAF1AL* gene was clearly elevated in the third whorl of both male and normal hermaphrodite flowers. Therefore, we could distinguish between the third whorl of normal and abnormal hermaphrodite flowers by identifying the differences in AS-seq and in the expression level of the *CpCAF1AL* gene (Figs 5–7 and S6 Table).

### Functional maps of the sex-related genes of *C*. *papaya* L.

We attempted to combine the results of sex-correlated genotyping and the specific patterns of expression/AS-seqs at the *CpSERK*, *CpSVPL*, and *CpCAF1AL* loci of the flowers of the different sex types, for functional mapping. The following results were observed in the third whorl at the *CpSERK*, *CpSVPL*, and *CpCAF1AL* loci, respectively: no variance/no AS-seq, high/no AS-seq, and high/no AS-seq expression for male flowers with XY^m^-type SNPs; no variance/no AS-seq, low/no AS-seq, and high/no AS-seq expression for normal hermaphrodite flowers with XY^h^-type SNPs; no variance/AS-seq, high/no AS-seq, and low/AS-seq expression for female sterile flowers (abnormal hermaphrodite flowers) with XY^h^-type SNPs; and no variance/AS-seq, low/no AS-seq, and low/AS-seq expression for carpellate flowers (abnormal hermaphrodite flowers) with XY^h^-type SNPs (Fig 7). Specifically, we found no variance/AS-seq, low/no AS-seq, but partial cDNA sequence, and low/AS-seq expression for the functional mapping in the fourth whorl of flowers with XX-type SNPs (Fig 7). Depending on the functional map of the flowers of different sex types, we found that the functional map in the third whorl of the normal hermaphrodite consisted of no variance/no AS-seqs without XX-type SNPs at the *CpSERK* locus; low/no AS-seqs with XY^h^-type SNPs at the *CpSVPL* locus; and high/no AS-seqs without XY^m^-type SNPs at the *CpCAF1AL* locus.

## Discussion

Papaya is a trioecious species with three sex types, i.e., female, male, and hermaphrodite. It is considered as an outstanding model system to study sex determination in plants. Because of recombination suppression, highly diverged sequences are present between the X and Y or Y^h^ chromosomes of papaya. According to the genetic hypothesis of sex determination, the combinations of dominant alleles, such as Y and Y^h^, are lethal to the zygote, signifying that the Y and Y^h^ chromosomes lack some genes that are essential for embryonic development [3]. MSY and hermaphrodite-specific region of Y^h^ (HSY) are extremely methylated and heterochromatized relative to their corresponding regions on the X chromosome [35]. These sequence alterations were used to develop DNA markers that are used for distinguishing between sex types in papaya. Gene marker systems, such as single-nucleotide polymorphisms (SNPs) and restriction fragment length polymorphisms (RFLPs), have been developed for gene discovery and mapping. SNPs are single-base differences that produce very dense genetic maps because of their abundance. SNP-based genetic maps were used to analyze the genome, including the performance of genome-wide linkage and association studies that assign genes to specific functions or traits [36].

In the present study, in addition to the occurrence of abnormal hermaphrodite flowers, we also found the occurrence of hermaphrodite flowers that developed at the ends of the inflorescences of male *C*. *papaya* L. plants. We propose that the related genes in the fourth whorl of pistils from the male plants of *C*. *papaya* L. were inhibited. Our results indicate that the expression level of the *CpSVPL* gene was high in the third whorl of male flowers and in the third whorl of female sterile flowers; however, the expression level of the *CpSVPL* gene was relatively low in the third and fourth whorls of the flowers that contained the fourth whorl structure (Fig 6). Based on our findings, we propose that *CpSVPL* may play a regulatory role in the development of the fourth whorl. In *A*. *thaliana*, plants that have mutations in both the *SVP* and *AGL24* genes exhibit decreased development of the fourth whorl of flowers in a high-temperature environment (>30°C) [16]. The papaya female sample (XX) showed no product in the intron 6 test of the RT–PCR assay (except for the intron 1 to 5 test results). However, in other sex types (XY), a band was present in nearly every sample (Fig 5). According to the qPCR analyses, the gene expression level was highest at junction 1 in the fourth whorl of female flowers. The expression levels at other junctions were extremely low (S7 Table). The findings of the present study regarding the *CpSVPL* gene were comparable to those of earlier reports by Urasaki *et al*. (2012) and Ueno H *et al*. (2015) regarding sex determination in papaya. A genome comparison study of male and hermaphrodite differentiation showed that *CpSVPL* is a Y- and Y^h^-chromosome-specific gene, as this allele is not present in the X chromosome [15, 37]. The Y and Y^h^ chromosomes carry different alleles of the *SVPL* gene; however, only the allele located in the Y chromosome encodes an intact protein with both the MADS-box and K-box domains. We propose that the gene expression level and mRNA sequence of the *CpSVPL* locus may differ substantially between the female and other sex types. These results suggest that amplification of the X chromosome occurred during its evolution in *C*. *papaya* L. [24, 38]. This amplification may also have led to differences in the sequences of the same gene in the Y and X chromosomes [23]. The NGS transcriptome results also seem to reflect this supposition reasonably (Fig 3A and S1 Dataset).

The genetic analysis of the sex-related genotypes of SNPs indicated that the sex-related SNPs are located in the *CpSVPL*, *CpSERK*, and *CpCAF1AL* genes in *C*. *papaya* L. These SNPs include SNPs in intron 3 (Fig 4A) and in the 5'-untranslated region (UTR) (NGS data not shown) of the *CpSVPL* gene, which showed AS in intron 4 (Fig 3A); SNPs in introns 1 and 7 and exon 9 (Fig 4B) of the *CpSERK* gene, which showed AS in introns 6, 7, and 8 (Fig 5B); and SNPs in exons 8 and 12 (Fig 4C) of the *CpCAF1AL* gene, which showed AS in intron 11 (Figs 3C and 5C). SNPs can affect AS in adjacent introns and in exons located 2–1000 bp upstream or downstream [39]. SNPs can also affect the stability and efficiency of mRNA transcription, which can lead to AS [40]. In the present study, sex-related SNPs were located in the range of sequences that affect AS.

Occurrences of AS were discovered in the *CpSERK* and *CpCAF1AL* genes in the fourth whorl of female flowers (XX) in this study; in contrast, the occurrence of AS was not observed in the third whorl of the male flowers (XY^m^) and normal hermaphrodite flowers (XY^h^). The same specific bands were observed in both sex types (Fig 5B and 5C). In a human gender study, the Y chromosome also exhibited specific expression in the testes, which are reproductive organs [25]. In fruit flies (*Drosophila melanogaster*), a link between AS-seqs and sex type was identified [41]. Different expression types of AS have also been identified in different organs in *A*. *thaliana* [42]. A higher level of expression of *AtSERK1* was noticed in closed flower buds before fertilization and in flowers with embryos at stages 1 through 7 after pollination [43]. In *Gossypium hirsutum*, *GhSERK1* expression was associated with the development of anthers [44]. Analysis of *GhSERK1* via RNAi studies revealed the presence of various levels of male sterility; higher levels of male sterility were observed in cases with lower expression levels of the *GhSERK1* mRNA [44]. In *Zea mays*, *ZmSERK1* was expressed in male and female reproductive organs, but showed higher expression in microspores [45]. Recently, various SERKs were identified in *Glycine max* by comparing the SNPs between wild and cultivated soybeans using genome-wide evolutionary analyses, which provided valuable tools for the identification of the functional divergence of this gene family [46].

CAF1 plays a role in the assembly of newly synthesized DNA for the formation of mature nucleosomes. CAF1 dysfunction causes a reduction in the heterochromatic fraction. The *CAF1A* gene may be related with X-chromosome inactivation and methylation of AS-seqs and DNA [30, 47]. A genome comparison sequence analysis performed in *C*. *papaya* L. showed that CAF1 is one of the uniquely expressed candidate genes with sequence polymorphism located in the MSY or HSY that are responsible for male or hermaphrodite determination [37]. In *A*. *thaliana*, the CAF1 pathway inactivity interrupts and arrests the cell cycle during pollen development [48]. In accordance with these studies, we propose that, without requiring AS-seqs, the normal development of male organs is supported by specific Y chromosome expression that may be caused by the specific and elevated expression of the *CpCAF1AL* gene in the third whorl.

**Fig 8:**
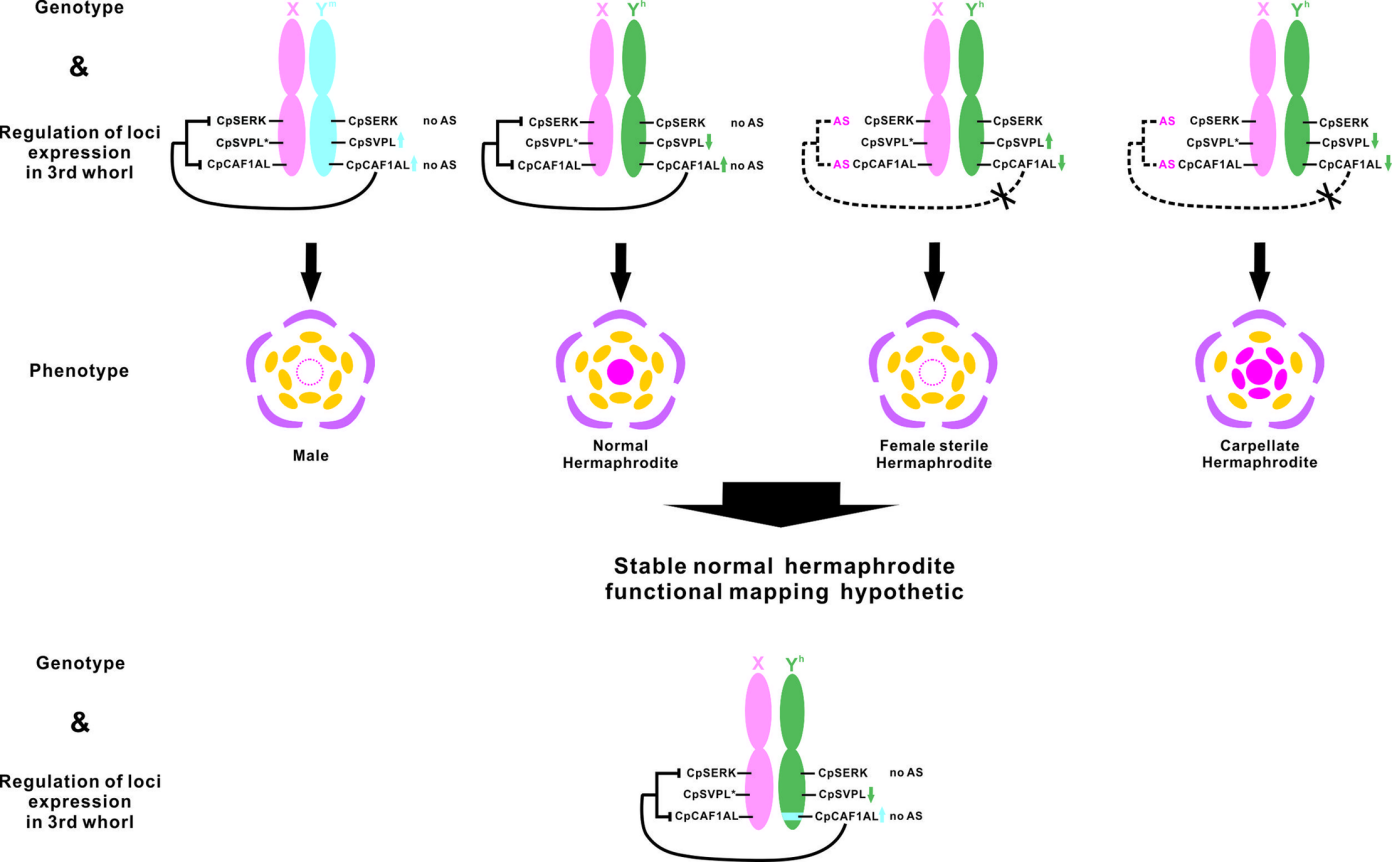
Hypothetic sex determination model involving the Y chromosome of *C*. *papaya* L. X: X chromosome; Y^m^: Y^m^ chromosome; Y^h^: Y^h^ chromosome; AS: AS-seq expression; blue arrows: male gene expression type; green arrows: hermaphrodite gene expression type; *: partial cDNA expression of the X chromosome; yellow ovals: third whorl of flowers; circles: fourth whorl of flowers; dotted line in circles: absence of the fourth whorl of flowers; fuchsia solid circles: carpellody tissues.

According to our results and those of the other studies discussed herein, we propose that sex-related SNPs may cause polymorphism of AS-seqs between X, Y^m^, and Y^h^ chromosomes. Hypothetically, at the *CpCAF1AL* locus, the pattern of AS-seq and low expression by the XX type may be replaced with the patterns of no AS-seq and high and stable expression by the XY^m^ type and high and unstable expression by the XY^h^ type, which may be related to the abnormal hermaphrodite flowers observed in the third whorl. Therefore, we suggest that the character of stable and normal hermaphrodite requires the *CpCAF1AL* locus with the XY^m^ type (Fig 8). Conversely, at the *CpSVPL* locus, we propose that the inhibition of the three sex types of flowers in the fourth whorl may be correlated with the specific patterns of expression of the SNPs of the XX type on the fourth whorl and the expression levels of the SNPs of the XY^m^ and XY^h^ types on the third whorl (Fig 8). Finally, at the *CpSERK* locus, we propose that the carpellate flowers may be correlated with the pattern of AS-seq on the third whorl, in cooperation with the low expression of the *CpSVPL* and *CpCAF1AL* loci and AS-seq of the *CpCAF1AL* loci in *C*. *papaya* L. (Fig 8).

## Conclusions

In *C*. *papaya* L., the sex types are determined by the pair of sex chromosomes. Moreover, the regions in the Y and Y^h^ chromosomes (designated as MSY and HSY regions, respectively) are difficult to study because of their intricate positioning, thus rendering the determination of males and hermaphrodites a difficult and complex process. Despite these issues, we performed a functional mapping of the three sex-related loci in different sex types and in the abnormal hermaphrodite flowers of *C*. *papaya* L. We also tried to perform functional mapping on the third whorl of the normal hermaphrodite flowers; however, there were no AS-seqs of *CpSERK*, the expression level of *CpSVPL* was low, and *CpCAF1AL* was expressed at high levels and lacked AS-seqs. The linkage maps of the stable normal hermaphrodite flowers, which were based on the sex-related SNP combinations, were also suggested (Fig 8). To identify the roles of these genes in sex determination, further functional analyses, including genetic transformation or mutation in papaya, are necessary. We also suggest that a genetic mapping analysis accompanying research of the stable normal hermaphrodite flowers of *C*. *papaya* L. remains necessary.

## Supporting information

S1 Dataset Sequence assembly and gene annotation from the transcriptome data of three sex types queried via the basic local alignment search tool (BLAST) against the 15 reference BACs of papaya. The datasets are compiled into an Excel file. AP1 AP4: 28 days before flowering hermaphrodite flower; AP2: 28 days before flowering female flower; AP3 and AP5: -28 days before flowering male flower.
(XLSX)

S1 Fig The gene functional maps from the transcriptome data of three sex types queried via BLAST against the 15 reference BACs of papaya. (A) the Y^h^ chromosome of BAC-49L11; (B) the X chromosome of BAC-50J21; (C) the Y^h^ chromosome of BAC-50M09; (D) the Y^h^ chromosome of BAC-53G04; (E) the Y chromosome of BAC-57M14; (F) the Y^h^ chromosome of BAC-62H24; (G) the Y^h^ chromosome of BAC-65D15; (H) the Y^h^ chromosome of BAC-71E16; (I) the Y^h^ chromosome of BAC-72J22; (J) the Y^h^ chromosome of BAC-81O12; (K) the Y^h^ chromosome of BAC-PH85B24; (L) the Y^h^ chromosome of BAC-90D06; (M) the Y chromosome of BAC-PH94E22; (N) the Y^h^ chromosome of BAC-PH95B12; (O) and the Y^h^ chromosome of BAC-96A24. The 15 reference BACs were used to estimate the locations, and the functional proteins are represented on the left side and the right side for each individual linkage. Following each protein function, the “>” sign links with the ID number in the database. The reference BACs represent the three sex type linkage populations. F: females; M: males; H: hermaphrodites; underlined and bold information indicates that the same functional protein exists in the three sex types; bold information indicates the same functional protein exists in two sex types.
(TIF)

S2 Fig The comparison among the three sex types of the *CpSVPL* gene from the Yh chromosome of BAC 71E16. (A) AA-seq comparison of the *CpSVPL* coding region; (B) Nucleotide sequence comparison of the *CpSVPL* cDNA of the three sex types.
(TIF)

S3 Fig The comparison among the three sex types of the *CpSERK* gene from the Yh chromosome of BAC 71E16. (A) AA-seq comparison of the *CpSERK* coding region; (B) Nucleotide sequence comparison of the *CpSERK* cDNA of the three sex types.
(TIF)

S4 Fig The comparison among the three sex types of the *CpCAF1AL* gene from the Yh chromosome of BAC 50M09. (A) AA-seq comparison of the *CpCAF1AL* coding region; (B) Nucleotide sequence comparison of the *CpCAF1AL* cDNA of the three sex types.
(TIF)

S5 Fig The normalized melting curve from the SNP-HRM assay for the three sex types of papaya. HRM curve assay results using the primers (A) CpSVPL_HRM_88; (B) CpSERK_HRM_30704; (C) CpSERK_HRM_34072; (D) CpSERK_HRM_34760; (E) CpSERK_HRM_34787; (F) CpCAF1AL_HRM_01; (G) CpCAF1AL_HRM_02; (H) CpCAF1AL_HRM_03; and (I) CpCAF1AL_HRM_04. F: females; M: males; H: hermaphrodites.
(TIF)

S6 Fig The melting peak of collective samples from the SNP-HRM assay for the three sex types of papaya. The melting peak of HRM analysis of (A) CpSVPL_HRM_88; (B) CpSERK_HRM_30704; (C) CpSERK_HRM_34072; (D) CpSERK_HRM_34787; (E) CpSERK_HRM_34787; (F) CpCAF1AL_HRM_01; (G) CpCAF1AL_HRM_02; and (H) CpCAF1AL_HRM_04 for collective samples. Sex sample tests included F: females, 5 samples; M: males, 3 samples; H: hermaphrodites, 4 samples. Tests also included blind samples. f: female melting peak; m: male melting peak e; h: hermaphrodite melting peak; mh: not female melting peak; fh: not male melting peak.
(TIF)

S7 Fig The PCR-RFLP analysis results of the sex-related SNPs of papaya. The analytical results of (A) CpSVPL_RFLP_Nde I; (B) CpSERK_RFLP_Spe I. M: marker (shown on the left side); H: hermaphrodites; FM: males of the Florida variety. The PCR products were digested using the restriction enzyme NdeI (shown behind the underline); the samples marked without an underline represent PCR products that were not subjected to restriction enzyme digestion; SF: females of Florida Variety; FM: males of the Florida variety; SH: hermaphrodites of Sunrise Solo. The PCR products were digested using the restriction enzyme Spe I (shown behind the underline).
(TIF)

S8 Fig Primer positions on the sex-related genes of papaya with respect to the RT-PCR and Q-PCR assays. (A) *CpSVPL*; (B) *CpSERK*; (C) *CpCAF1AL* genes. ‘●’: start codon; ‘▲’: stop codon; black thin line: intron of gene; I#: number of introns; gray thick line: exon of gene; E#: number of exons; underline: primer name.
(TIF)

S9 Fig The RT-PCR assay results of the *CpSVPL* gene. (A) Intron 1; (B) Intron 2; (C) Intron 3; (D) Intron 4; (E) Intron 5; (F) Intron 6 test. Arrows indicate the RT-PCR product sizes (in bp) from cDNA after normal splicing. M: marker; 1: 28 days before flowering female buds; 2: 28 days before flowering male buds; 3: 28 days before flowering normal hermaphrodite buds; 4: 28 female degradation hermaphrodite buds; 5: 28 days before flowering carpellody hermaphrodite buds; 6: female fourth whorl; 7: male third whorl; 8: normal hermaphrodite fourth whorl; 9: normal hermaphrodite third whorl; 10: female degradation hermaphrodite third whorl; 11: carpellody hermaphrodite fourth whorl; 12: carpellody hermaphrodite third whorl. B: blank.
(TIF)

S10 Fig The RT-PCR assay results of the *CpSERK* gene. (A) Intron 1; (B) Intron 2; (C) Intron 3; (D) Intron 4; (E) Intron 5; (F) Intron 6; (G) Intron 7; (H) Intron 8; (I) Intron 9; (J) Intron 10 test. Arrows indicate the RT-PCR product sizes (in bp) from cDNA after normal splicing. M: marker; 1: 28 days before flowering female buds; 2: 28 days before flowering male buds; 3: 28 days before flowering normal hermaphrodites buds; 4: 28days before flowering female degradation hermaphrodite buds; 5: 28 days before flowering carpellody hermaphrodite buds; 6: female fourth whorl; 7: male third whorl; 8: normal hermaphrodite fourth whorl; 9: normal hermaphrodite third whorl; 10: female degradation hermaphrodite third whorl; 11: carpellody hermaphrodite fourth whorl; 12: carpellody hermaphrodite third whorl. B: blank.
(TIF)

S11 Fig The RT-PCR assay results of the *CpCAF1AL* gene. (A) Intron 1; (B) Intron 2; (C) Intron 3; (D) Intron 4; (E) Intron 5; (F) Intron 6; (G) Intron 7; (H) Intron 8; (I) Intron 9; (J) Intron 10; (K) Intron 11 test. Arrows indicate the RT-PCR product sizes (in bp) from cDNA after normal splicing. M: marker; 1: 28 days before flowering female buds; 2: 28 days before flowering male buds; 3: 28 days before flowering normal hermaphrodite buds; 4: 28 days before flowering female degradation hermaphrodite buds; 5: 28 days before flowering carpellody hermaphrodite buds; 6: female fourth whorl; 7: male third whorl; 8: normal hermaphrodite fourth whorl; 9: normal hermaphrodite third whorl; 10: female degradation hermaphrodite third whorl; 11: carpellody hermaphrodite fourth whorl; 12: carpellody hermaphrodite third whorl. B: blank.
(TIF)

S12 Fig The electrophoresis results of the Q-PCR products of the *CpSVPL* gene. (A) Junction 1; (B) Junction 2; (C) Junction 3; (D) Junction 4; (E) Junction 5; (F) Junction 6. Arrows indicate the Q-PCR product sizes (in bp). M: marker; 1: 28 days before flowering female buds; 2: 28 days before flowering male buds; 3: 28 days before flowering normal hermaphrodite buds; 4: 28 days before flowering female degradation hermaphrodite buds; 5: 28 days before flowering carpellody hermaphrodite buds; 6: female fourth whorl; 7: male third whorl; 8: normal hermaphrodite fourth whorl; 9: normal hermaphrodite third whorl; 10: female degradation hermaphrodite third whorl; 11: carpellody hermaphrodite fourth whorl; 12: carpellody hermaphrodite third whorl.
(TIF)

S13 Fig The electrophoresis results of the Q-PCR products of the *CpSERK* gene. (A) Junction 1; (B) Junction 2; (C) Junction 3; (D) Junction 4; (E) Junction 5; (F) Junction 6; (G) Junction 7; (H) Junction 8; (I) Junction 9; (J) Junction 10. Arrows indicate the Q-PCR product sizes (in bp). M: marker; 1: 28 days before flowering female buds; 2: 28 days before flowering male buds; 3: 28 days before flowering normal hermaphrodite buds; 4: 28 days before flowering female degradation hermaphrodite buds; 5: 28 days before flowering carpellody hermaphrodite buds; 6: female fourth whorl; 7: male third whorl; 8: normal hermaphrodite fourth whorl; 9: normal hermaphrodite third whorl; 10: female degradation hermaphrodite third whorl; 11: carpellody hermaphrodite fourth whorl; 12: carpellody hermaphrodite third whorl.
(TIF)

S14 Fig The electrophoresis results of the Q-PCR products of the *CpCAF1AL* gene. (A) Junction 1; (B) Junction 2; (C) Junction 3; (D) Junction 4; (E) Junction 5; (F) Junction 6; (G) Junction 7; (H) Junction 8; (I) Junction 9; (J) Junction 10; (K) Junction 11. Arrows indicate the Q-PCR product sizes (in bp). M: marker; 1: 28 days before flowering female buds; 2: 28 days before flowering male buds; 3: 28 days before flowering normal hermaphrodite buds; 4: 28 days before flowering female degradation hermaphrodite buds; 5: 28 days before flowering carpellody hermaphrodite buds; 6: female fourth whorl; 7: male third whorl; 8: normal hermaphrodite fourth whorl; 9: normal hermaphrodite third whorl; 10: female degradation hermaphrodite third whorl; 11: carpellody hermaphrodite fourth whorl; 12: carpellody hermaphrodite third whorl.
(TIF)

S1 Table The gene numbers of the different sex groupings from the transcriptome data of the three sex types refer to the 15 BACs of papaya. (DOCX)

S2 Table The gene numbers of the different AS-seq types from the transcriptome data of the three sex types refer to the 15 BACs of papaya. (DOCX)

S3 Table The gene numbers of the different sex groupings and AS-seq types from the transcriptome data of the three sex types refer to the 15 BACs of papaya. type_1: the genes are not expressed in one of the three sex types, and there are differences in their AA-seqs between those of the other two sex types; type_2: the genes are expressed in all three sex types, and there are differences in their AA-seqs; type_3: the genes are expressed in all three sex types, and there is no difference in AA-seqs between two sex types.
(DOCX)

S4 Table Genotyping of three sex types, primer name and primer sequence for SNP-HRM assay on the sex-related genes of papaya. (DOCX)

S5 Table The primer name and sequence for RT-PCR and Q-PCR assays of the sex-related genes of papaya. (DOCX)

S6 Table The summary of the RT-PCR and Q-PCR analysis results of the flowers of different sex types in the fourth and third whorls of flowers. 28 days before flowering: different sex types at 28 days before flowering; 0 days before flowering: different sex types at 0 days before flowering; ND: not determined; -: no difference; x: no primer design; F: different female; M: different male; H: different hermaphrodite; HM3: different female degradation hermaphrodite; 4: fourth whorl of flower; 3: third whorl of flower.
(DOCX)

S7 Table The average expression of each junction of the CpSVPL gene based on the results of the Q-PCR assay using three sample repeats of twelve samples. Gray area: lowest expression junction of the *CpSVPL* gene.
(DOCX)

S8 Table The average expression of each junction of the *CpSERK* gene based on the results of the Q-PCR assay using three sample repeats of twelve samples. Gray area: lowest expression junction of the *CpSERK* gene.
(DOCX)

S9 Table The average expression of each junction of the *CpCAF1AL* gene based on the results of the Q-PCR assay using three sample repeats of twelve samples. Gray area: lowest expression junction of the *CpCAF1AL* gene.
(DOCX)
