# Correction: The development of functional mapping by three sex-related loci on the third whorl of different sex types of *Carica papaya* L.

**DOI:** 10.1371/journal.pone.0196789

**Published:** 2018-04-26

**Authors:** Chen-Yu Lee, Hui-Jun Lin, Kotapati Kasi Viswanath, Chih-Peng Lin, Bill Chia-Han Chang, Pei-Hsun Chiu, Chan-Tai Chiu, Ren-Huang Wang, Shih-Wen Chin, Fure-Chyi Chen

There is an error in Table 1. The values in the third column "SNP position" are listed incorrectly. Please see the corrected Table 1 here.

**Table 1:** The sex correlation percentage and chi-squared analysis results.

SNP related sex type	BAC name	SNP position	Correlation (%)			Blind sample HRM test				
			F	H	M	F	H	M	Sex ratio	Square
3 sex type	Y^h^ chromosome BAC-71E16	8511	100%	100%	100%	31	31	30	1:1:1	0.01
Female type	Y^h^ chromosome BAC-71E16	122453	100%	94%	93%	42	54 (M + H)		1:2	4.69*
	Y^h^ chromosome BAC-71E16	119290, 119295	100%	100%	71%	26	54 (M + H)		1:2	0.02
	Y^h^ chromosome BAC-71E16	118602	100%	100%	67%	38	58 (M + H)		1:2	1.69
	Y^h^ chromosome BAC-71E16	118575	100%	100%	47%	35	61 (M + H)		1:2	0.42
Male type	Y^h^ chromosome BAC-50M09	144693	83%	83%	100%	76 (F + H)		35	2:1	0.16
	Y^h^ chromosome BAC-50M09	144603	83%	86%	100%	60 (F + H)		26	2:1	4.08*
	Y^h^ chromosome BAC-50M09	148090	100%	86%	100%	68 (F + H)		40	2:1	0.67

The progenies of male and hermaphrodite parental crosses from the SNP-HRM assay of the three sex types of papaya.

* Significance at *P*< 0.05.
