# Supplementary figures and images for: The development of functional mapping by three sex-related loci on the third whorl of different sex types of Carica papaya L
Source: PLoS One. 2018 Mar 22;13(3):e0194605. doi: 10.1371/journal.pone.0194605 (PMC5864051; doi:10.1371/journal.pone.0194605)

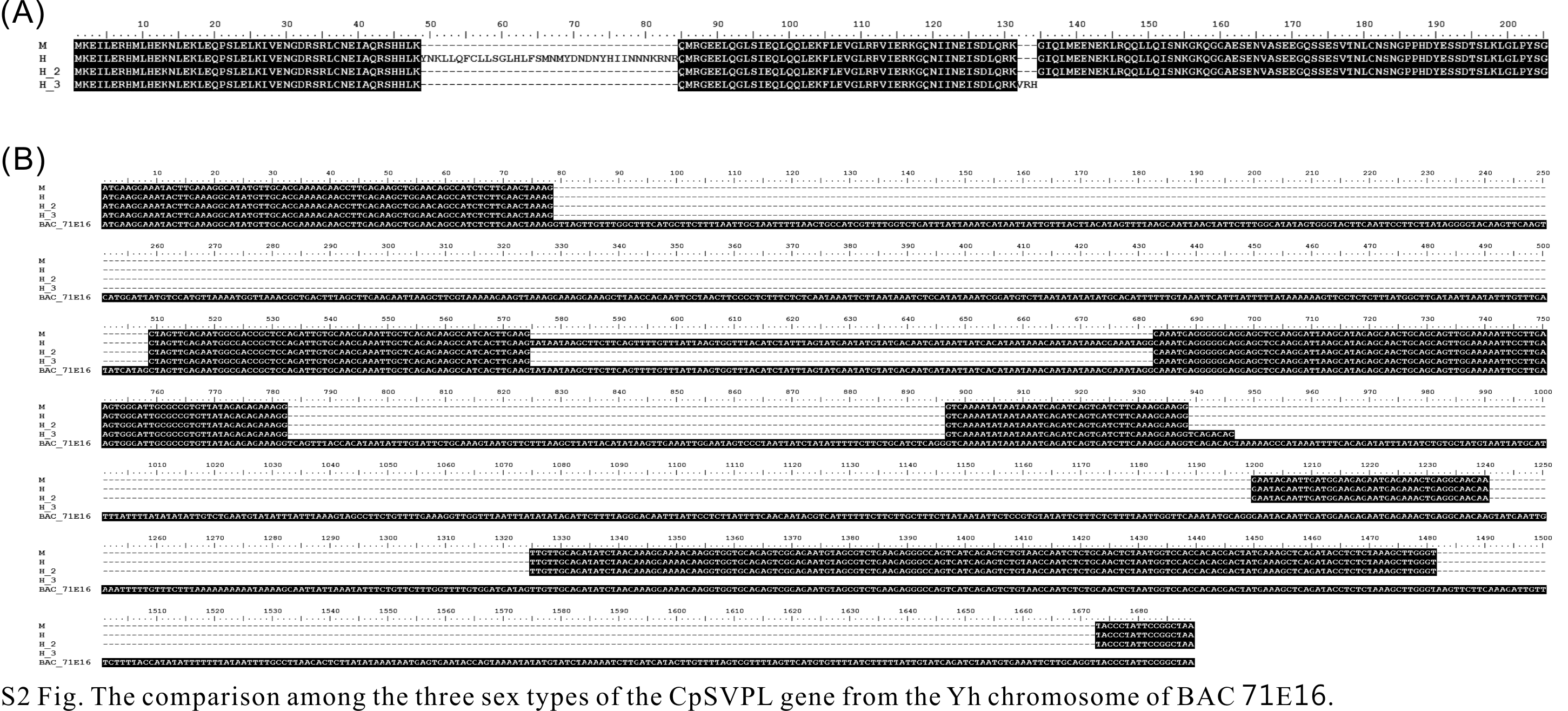

Supplement: S2 Fig — (A) AA-seq comparison of the CpSVPL coding region; (B) Nucleotide sequence comparison of the CpSVPL cDNA of the three sex types. (TIF) [file pone.0194605.s003.tif]

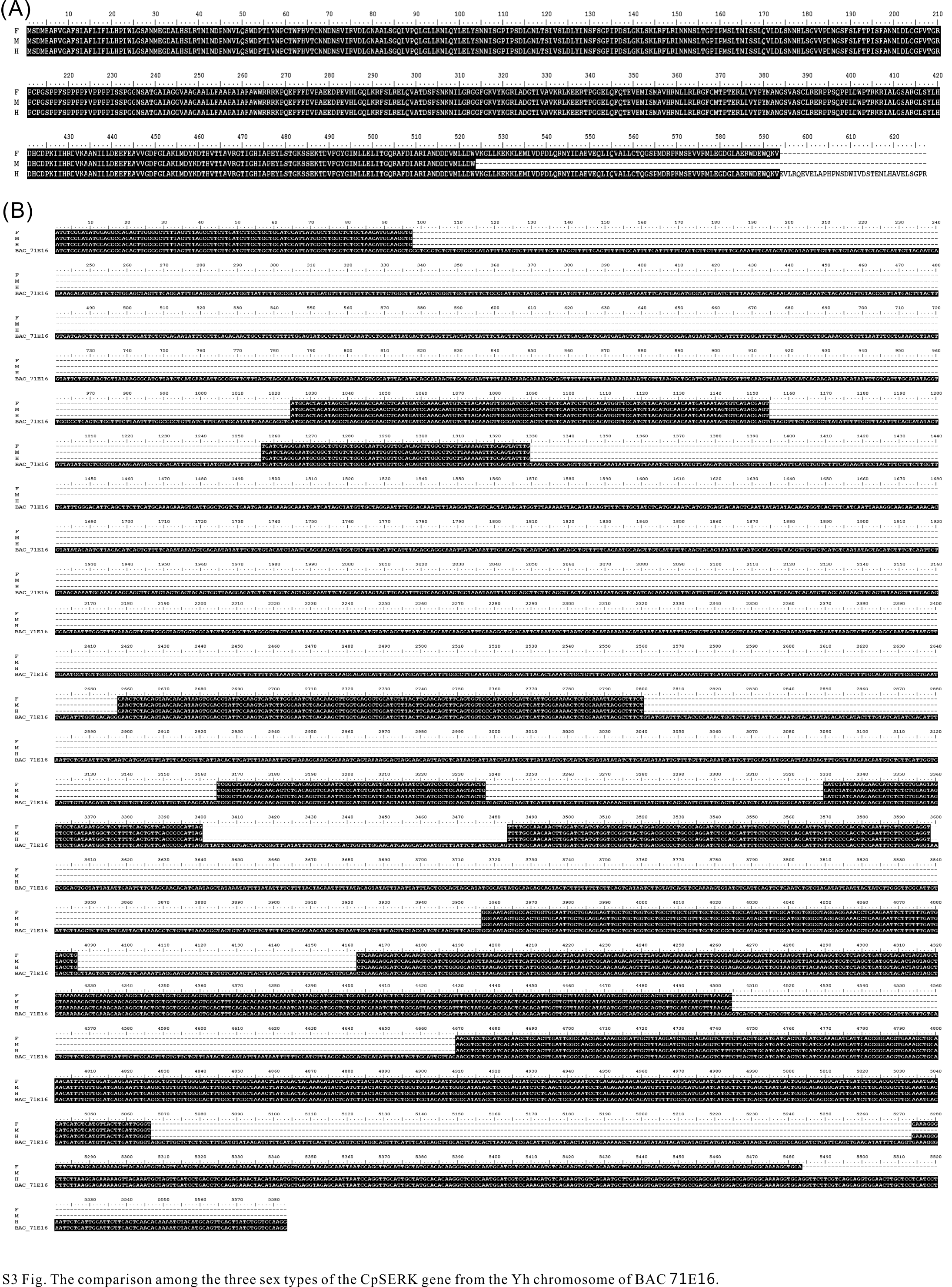

Supplement: S3 Fig — (A) AA-seq comparison of the CpSERK coding region; (B) Nucleotide sequence comparison of the CpSERK cDNA of the three sex types. (TIF) [file pone.0194605.s004.tif]

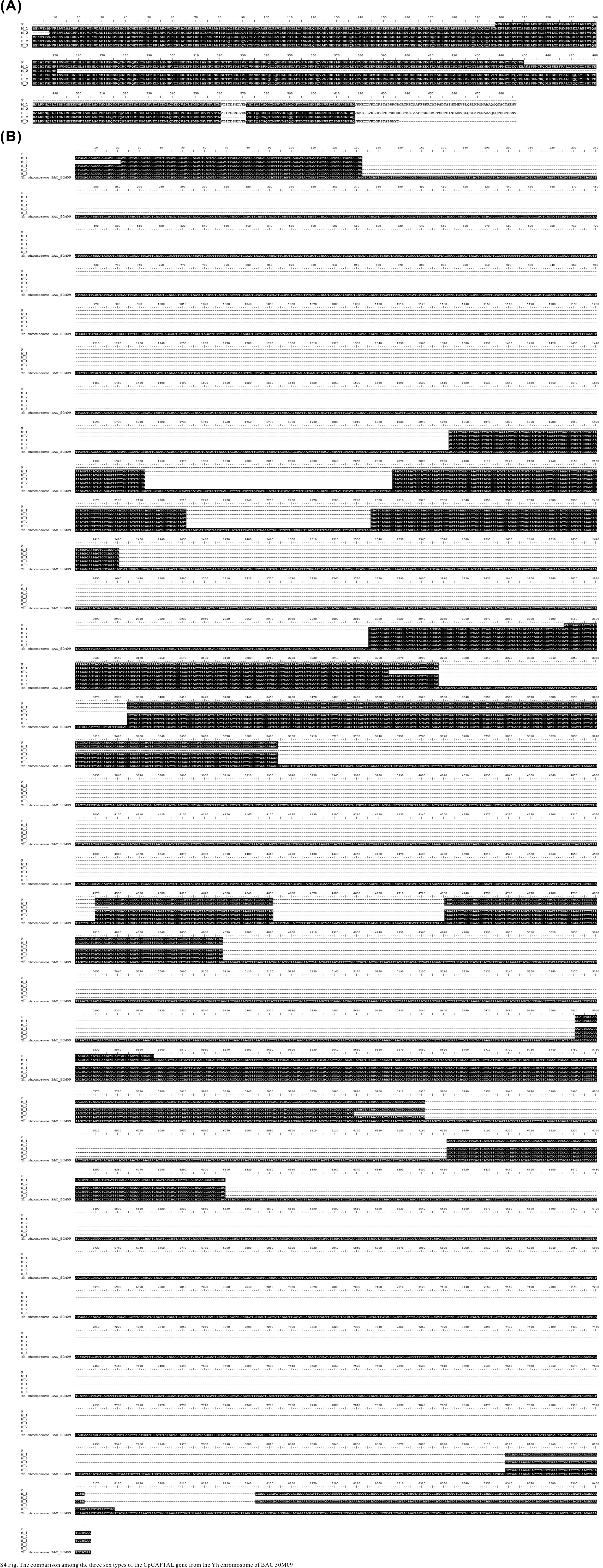

Supplement: S4 Fig — (A) AA-seq comparison of the CpCAF1AL coding region; (B) Nucleotide sequence comparison of the CpCAF1AL cDNA of the three sex types. (TIF) [file pone.0194605.s005.tif]

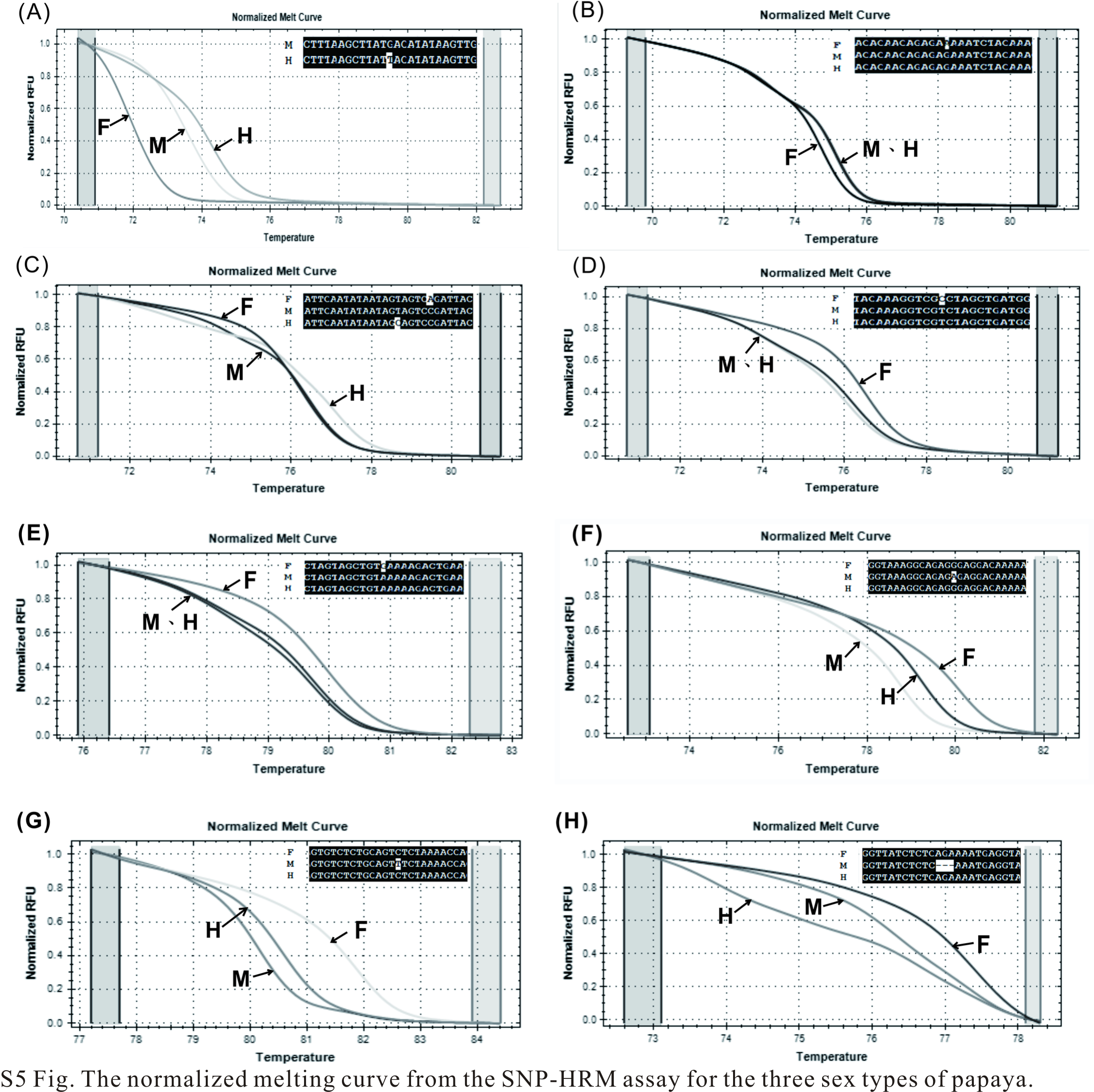

Supplement: S5 Fig — HRM curve assay results using the primers (A) CpSVPL_HRM_88; (B) CpSERK_HRM_30704; (C) CpSERK_HRM_34072; (D) CpSERK_HRM_34760; (E) CpSERK_HRM_34787; (F) CpCAF1AL_HRM_01; (G) CpCAF1AL_HRM_02; (H) CpCAF1AL_HRM_03; and (I) CpCAF1AL_HRM_04. F: females; M: males; H: hermaphrodites. (TIF) [file pone.0194605.s006.tif]

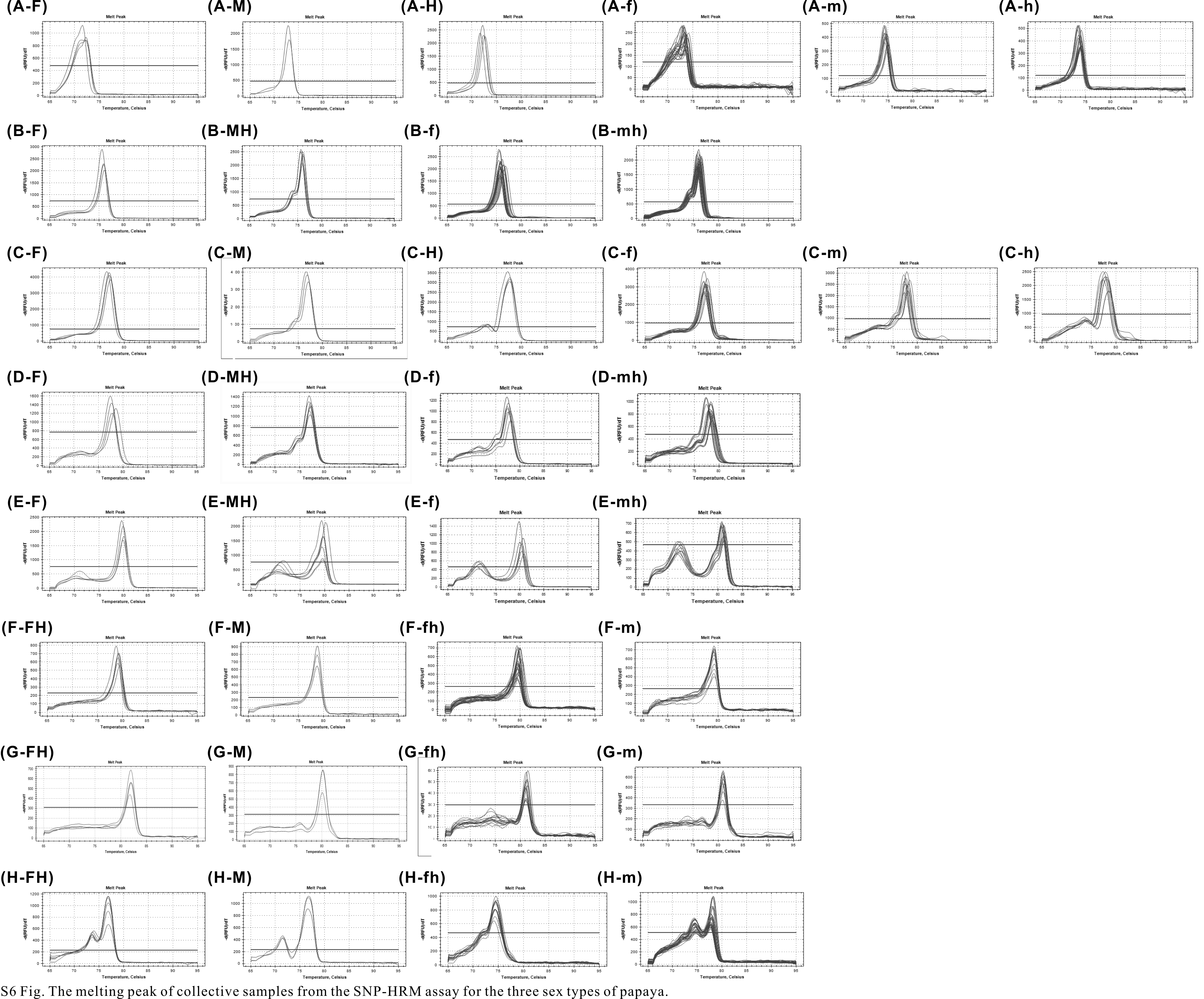

Supplement: S6 Fig — The melting peak of HRM analysis of (A) CpSVPL_HRM_88; (B) CpSERK_HRM_30704; (C) CpSERK_HRM_34072; (D) CpSERK_HRM_34787; (E) CpSERK_HRM_34787; (F) CpCAF1AL_HRM_01; (G) CpCAF1AL_HRM_02; and (H) CpCAF1AL_HRM_04 for collective samples. Sex sample tests included F: females, 5 samples; M: males, 3 samples; H: hermaphrodites, 4 samples. Tests also included blind samples. f: female melting peak; m: male melting peak e; h: hermaphrodite melting peak; mh: not female melting peak; fh: not male melting peak. (TIF) [file pone.0194605.s007.tif]

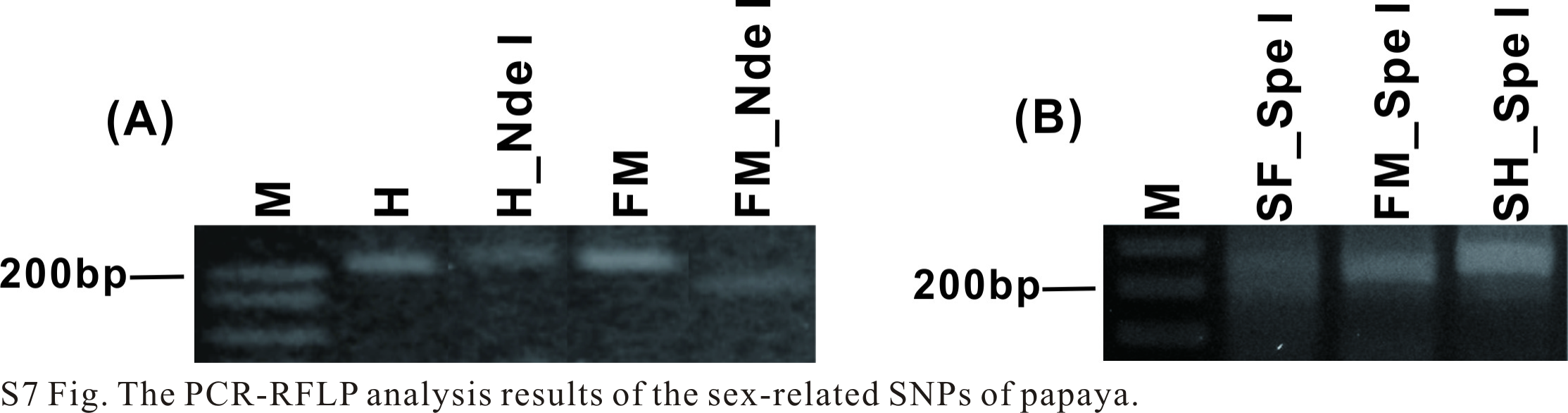

Supplement: S7 Fig — The analytical results of (A) CpSVPL_RFLP_Nde I; (B) CpSERK_RFLP_Spe I. M: marker (shown on the left side); H: hermaphrodites; FM: males of the Florida variety. The PCR products were digested using the restriction enzyme NdeI (shown behind the underline); the samples marked without an underline represent PCR products that were not subjected to restriction enzyme digestion; SF: females of Florida Variety; FM: males of the Florida variety; SH: hermaphrodites of Sunrise Solo. The PCR products were digested using the restriction enzyme Spe I (shown behind the underline). (TIF) [file pone.0194605.s008.tif]

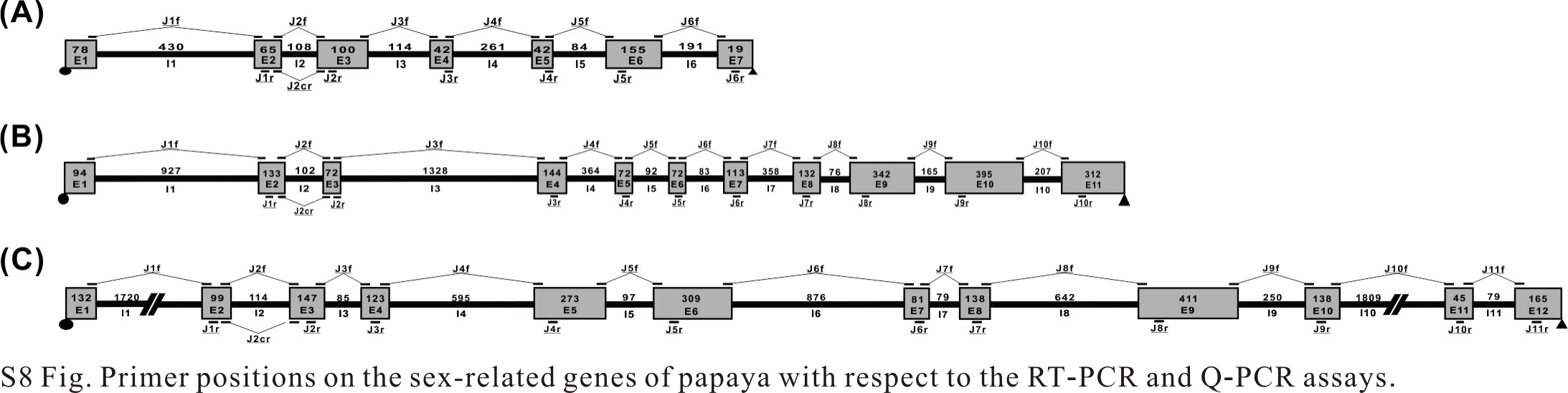

Supplement: S8 Fig — (A) CpSVPL; (B) CpSERK; (C) CpCAF1AL genes. ‘●’: start codon; ‘▲’: stop codon; black thin line: intron of gene; I#: number of introns; gray thick line: exon of gene; E#: number of exons; underline: primer name. (TIF) [file pone.0194605.s009.tif]

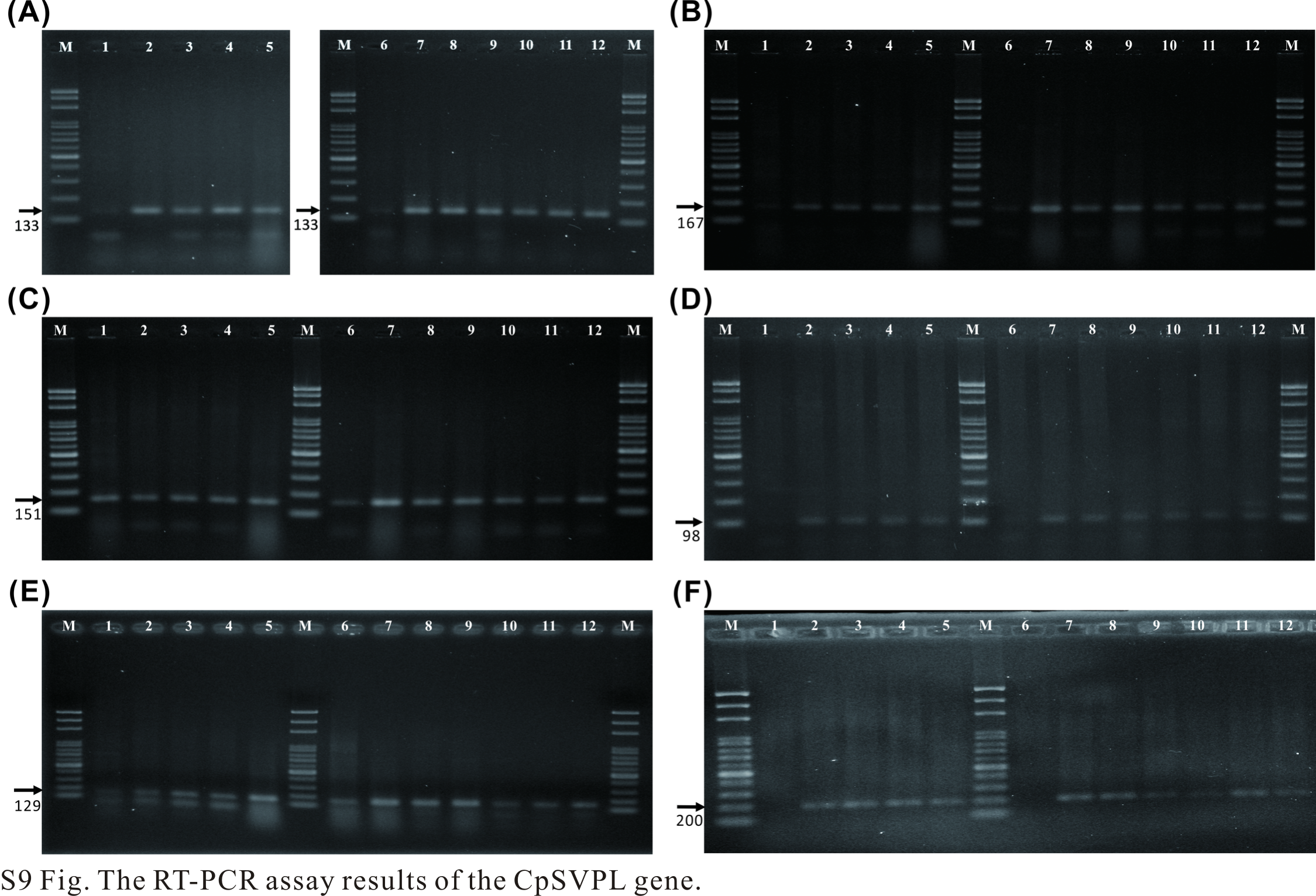

Supplement: S9 Fig — (A) Intron 1; (B) Intron 2; (C) Intron 3; (D) Intron 4; (E) Intron 5; (F) Intron 6 test. Arrows indicate the RT-PCR product sizes (in bp) from cDNA after normal splicing. M: marker; 1: 28 days before flowering female buds; 2: 28 days before flowering male buds; 3: 28 days before flowering normal hermaphrodite buds; 4: 28 female degradation hermaphrodite buds; 5: 28 days before flowering carpellody hermaphrodite buds; 6: female fourth whorl; 7: male third whorl; 8: normal hermaphrodite fourth whorl; 9: normal hermaphrodite third whorl; 10: female degradation hermaphrodite third whorl; 11: carpellody hermaphrodite fourth whorl; 12: carpellody hermaphrodite third whorl. B: blank. (TIF) [file pone.0194605.s010.tif]

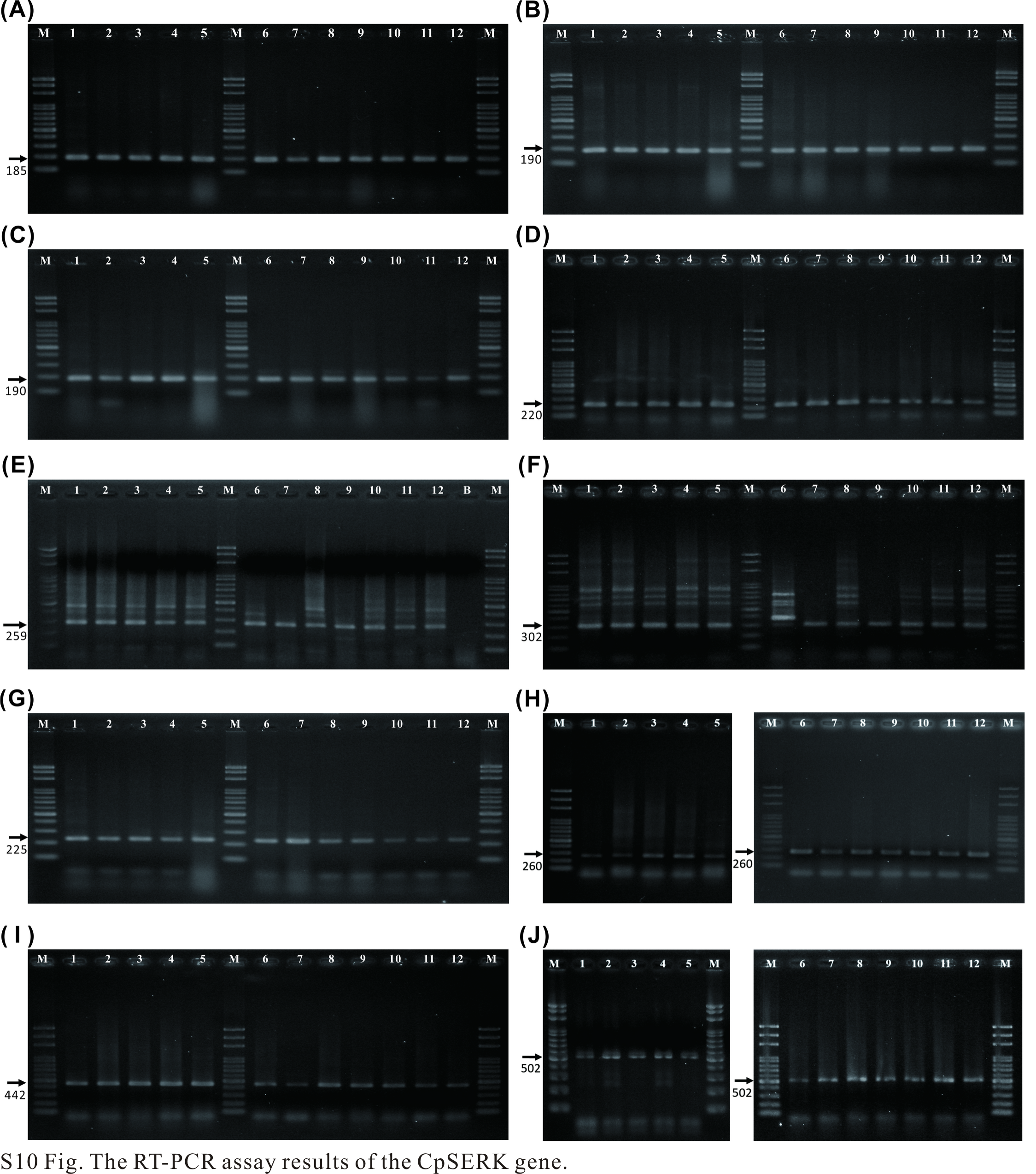

Supplement: S10 Fig — (A) Intron 1; (B) Intron 2; (C) Intron 3; (D) Intron 4; (E) Intron 5; (F) Intron 6; (G) Intron 7; (H) Intron 8; (I) Intron 9; (J) Intron 10 test. Arrows indicate the RT-PCR product sizes (in bp) from cDNA after normal splicing. M: marker; 1: 28 days before flowering female buds; 2: 28 days before flowering male buds; 3: 28 days before flowering normal hermaphrodites buds; 4: 28days before flowering female degradation hermaphrodite buds; 5: 28 days before flowering carpellody hermaphrodite buds; 6: female fourth whorl; 7: male third whorl; 8: normal hermaphrodite fourth whorl; 9: normal hermaphrodite third whorl; 10: female degradation hermaphrodite third whorl; 11: carpellody hermaphrodite fourth whorl; 12: carpellody hermaphrodite third whorl. B: blank. (TIF) [file pone.0194605.s011.tif]

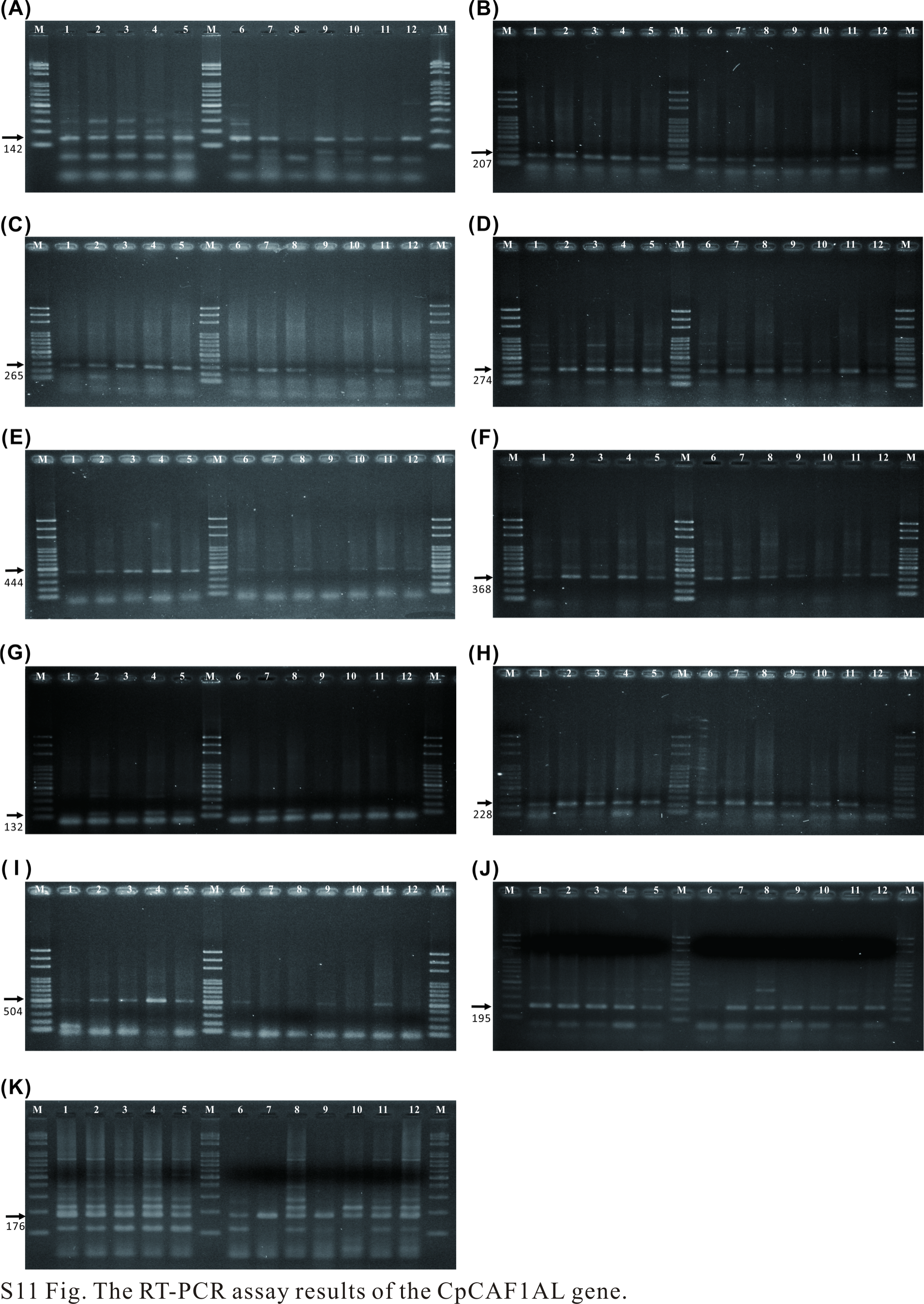

Supplement: S11 Fig — (A) Intron 1; (B) Intron 2; (C) Intron 3; (D) Intron 4; (E) Intron 5; (F) Intron 6; (G) Intron 7; (H) Intron 8; (I) Intron 9; (J) Intron 10; (K) Intron 11 test. Arrows indicate the RT-PCR product sizes (in bp) from cDNA after normal splicing. M: marker; 1: 28 days before flowering female buds; 2: 28 days before flowering male buds; 3: 28 days before flowering normal hermaphrodite buds; 4: 28 days before flowering female degradation hermaphrodite buds; 5: 28 days before flowering carpellody hermaphrodite buds; 6: female fourth whorl; 7: male third whorl; 8: normal hermaphrodite fourth whorl; 9: normal hermaphrodite third whorl; 10: female degradation hermaphrodite third whorl; 11: carpellody hermaphrodite fourth whorl; 12: carpellody hermaphrodite third whorl. B: blank. (TIF) [file pone.0194605.s012.tif]

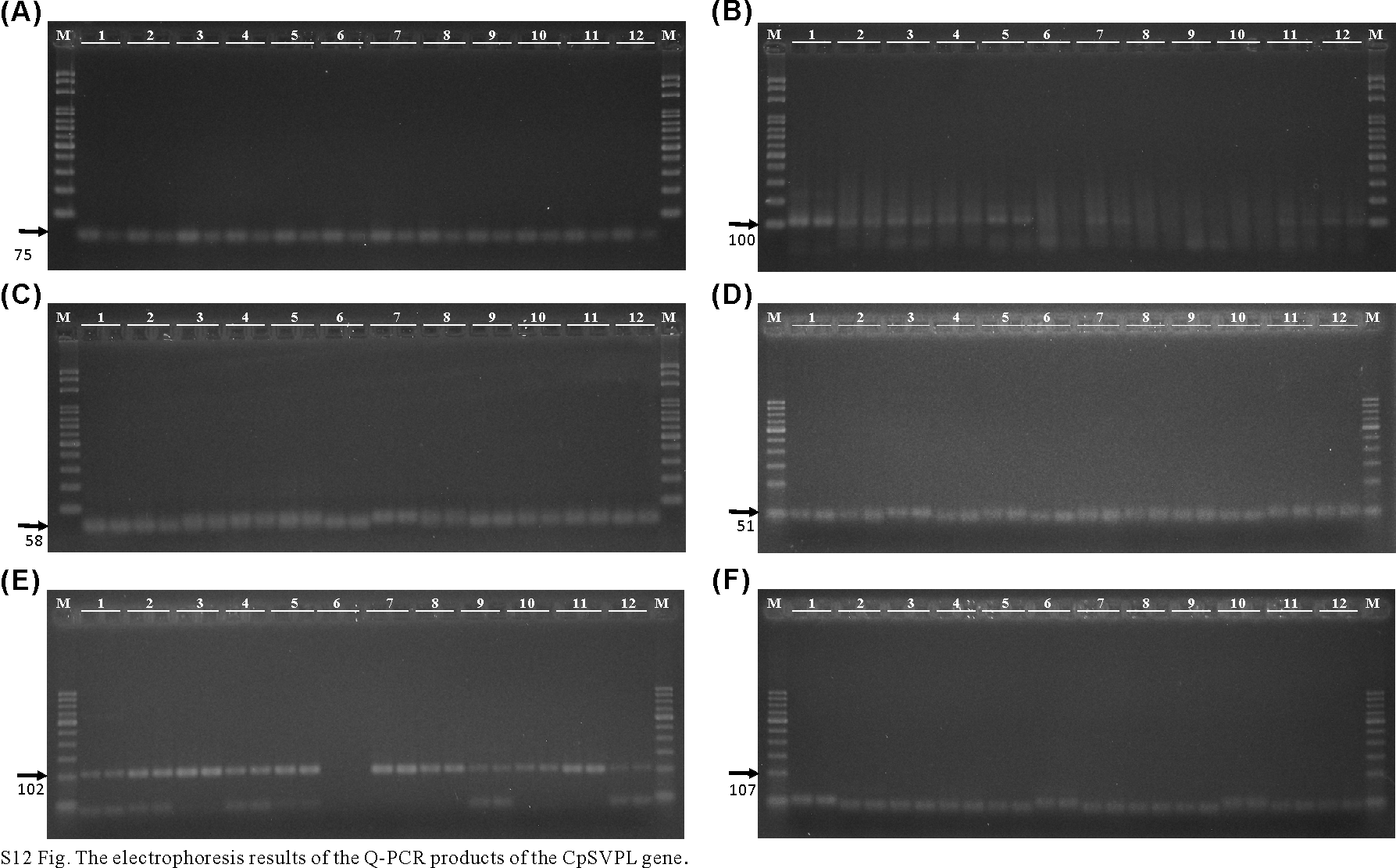

Supplement: S12 Fig — (A) Junction 1; (B) Junction 2; (C) Junction 3; (D) Junction 4; (E) Junction 5; (F) Junction 6. Arrows indicate the Q-PCR product sizes (in bp). M: marker; 1: 28 days before flowering female buds; 2: 28 days before flowering male buds; 3: 28 days before flowering normal hermaphrodite buds; 4: 28 days before flowering female degradation hermaphrodite buds; 5: 28 days before flowering carpellody hermaphrodite buds; 6: female fourth whorl; 7: male third whorl; 8: normal hermaphrodite fourth whorl; 9: normal hermaphrodite third whorl; 10: female degradation hermaphrodite third whorl; 11: carpellody hermaphrodite fourth whorl; 12: carpellody hermaphrodite third whorl. (TIF) [file pone.0194605.s013.tif]

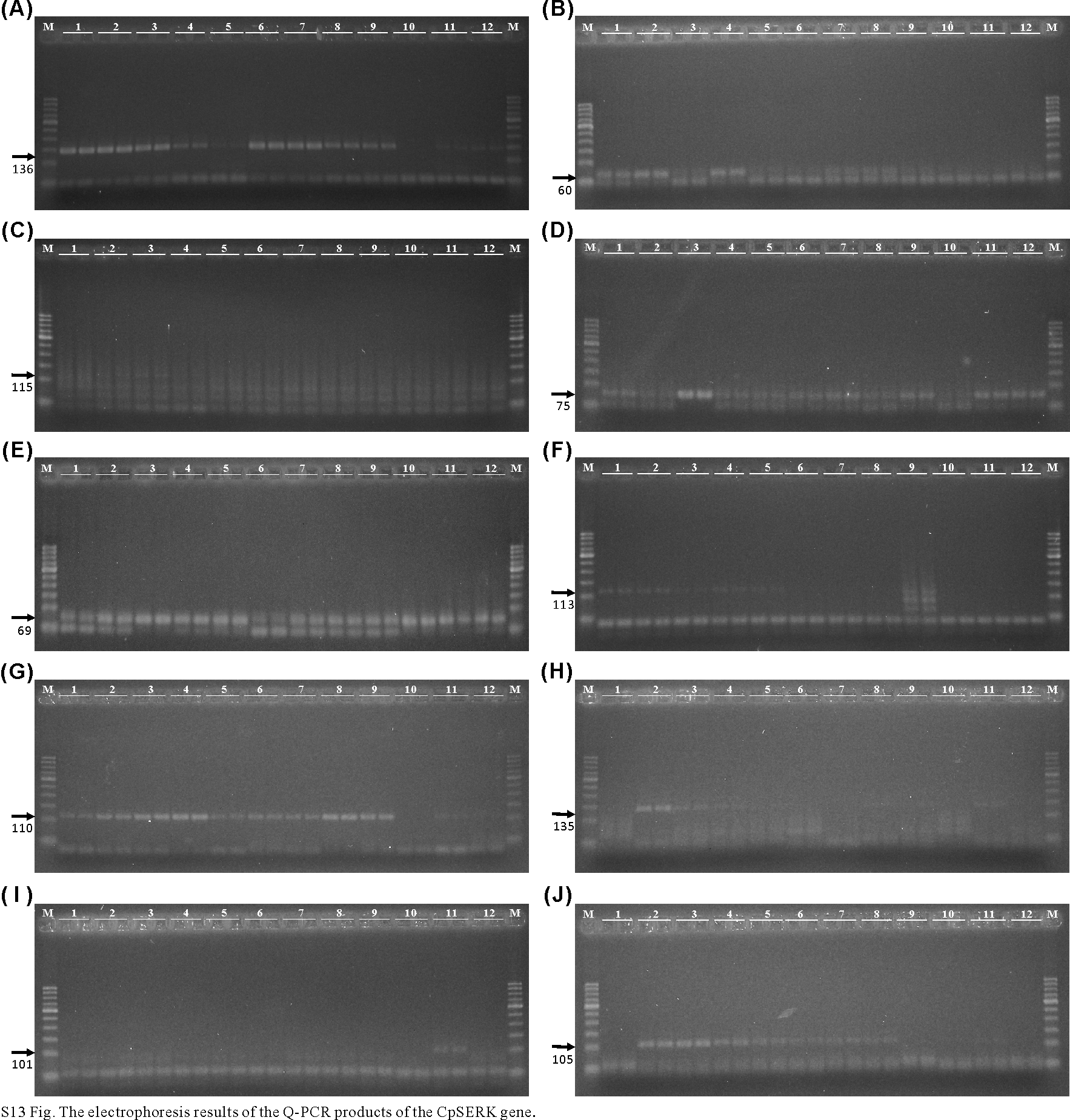

Supplement: S13 Fig — (A) Junction 1; (B) Junction 2; (C) Junction 3; (D) Junction 4; (E) Junction 5; (F) Junction 6; (G) Junction 7; (H) Junction 8; (I) Junction 9; (J) Junction 10. Arrows indicate the Q-PCR product sizes (in bp). M: marker; 1: 28 days before flowering female buds; 2: 28 days before flowering male buds; 3: 28 days before flowering normal hermaphrodite buds; 4: 28 days before flowering female degradation hermaphrodite buds; 5: 28 days before flowering carpellody hermaphrodite buds; 6: female fourth whorl; 7: male third whorl; 8: normal hermaphrodite fourth whorl; 9: normal hermaphrodite third whorl; 10: female degradation hermaphrodite third whorl; 11: carpellody hermaphrodite fourth whorl; 12: carpellody hermaphrodite third whorl. (TIF) [file pone.0194605.s014.tif]

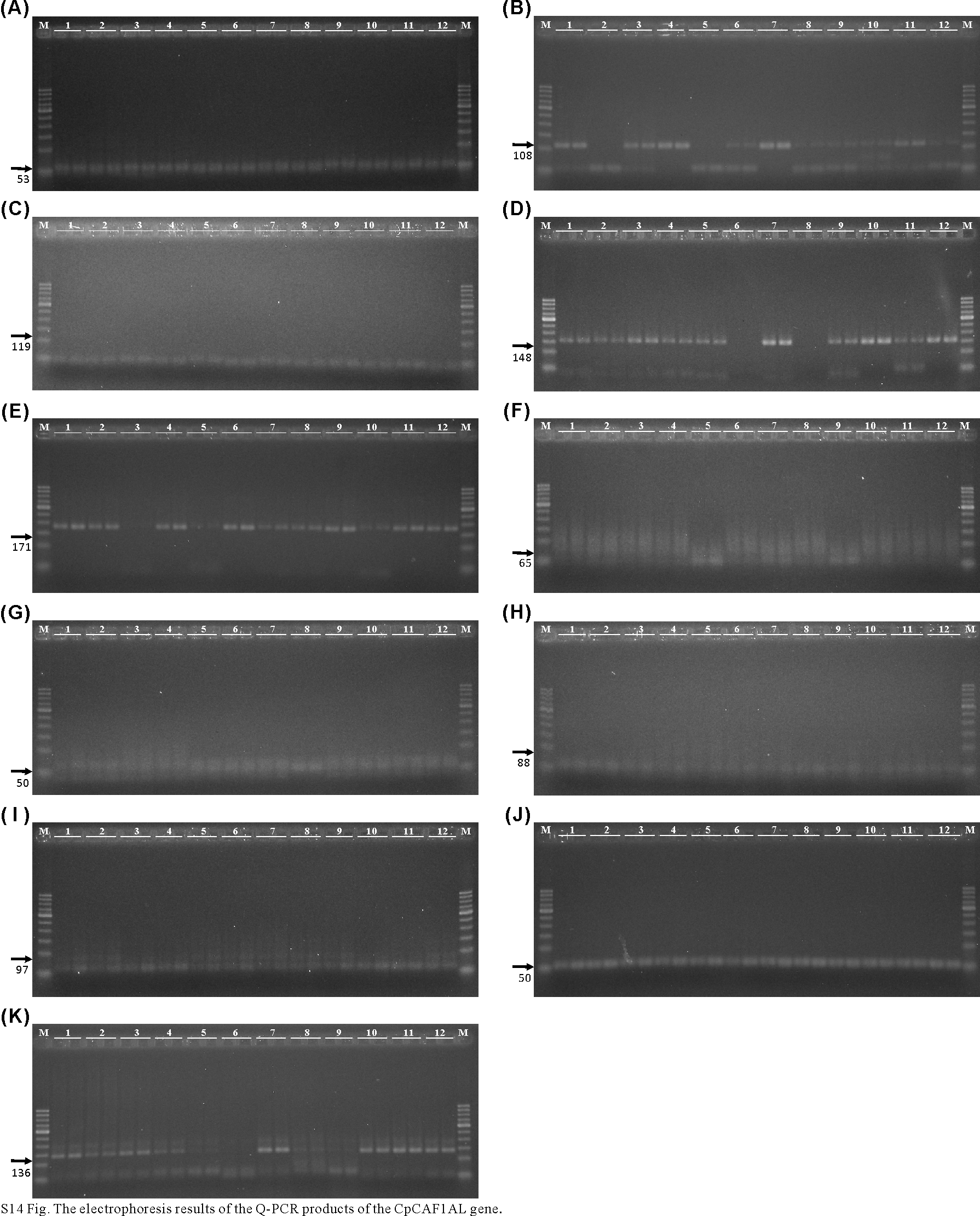

Supplement: S14 Fig — (A) Junction 1; (B) Junction 2; (C) Junction 3; (D) Junction 4; (E) Junction 5; (F) Junction 6; (G) Junction 7; (H) Junction 8; (I) Junction 9; (J) Junction 10; (K) Junction 11. Arrows indicate the Q-PCR product sizes (in bp). M: marker; 1: 28 days before flowering female buds; 2: 28 days before flowering male buds; 3: 28 days before flowering normal hermaphrodite buds; 4: 28 days before flowering female degradation hermaphrodite buds; 5: 28 days before flowering carpellody hermaphrodite buds; 6: female fourth whorl; 7: male third whorl; 8: normal hermaphrodite fourth whorl; 9: normal hermaphrodite third whorl; 10: female degradation hermaphrodite third whorl; 11: carpellody hermaphrodite fourth whorl; 12: carpellody hermaphrodite third whorl. (TIF) [file pone.0194605.s015.tif]
