# Supplementary material for: The development of functional mapping by three sex-related loci on the third whorl of different sex types of Carica papaya L
Source: PLoS One. 2018 Mar 22;13(3):e0194605. doi: 10.1371/journal.pone.0194605 (PMC5864051; doi:10.1371/journal.pone.0194605)
Supplement: S1 Table — (DOCX) [file pone.0194605.s016.docx]

Supplementary Table 1. The gene numbers of the different sex groupings from the transcriptome data of the three sex types refer to the 15 BACs of papaya.

| Papaya_BAC_ID | single sex expression types | | | Two sex expression types | | | Three sex expression type | Total of gene # |
| --- | --- | --- | --- | --- | --- | --- | --- | --- |
|  | F | M | H | F/M | F/H | M/H | F/M/H |  |
| Y^h^ chromosome BAC_49L11 | 1 | 2 | 1 | 0 | 0 | 1 | 1 | 6 |
| X chromosome BAC_50J21 | 5 | 2 | 4 | 1 | 2 | 4 | 10 | 28 |
| Y^h^ chromosome BAC_50M09 | 2 | 0 | 0 | 0 | 0 | 0 | 1 | 3 |
| Y^h^ chromosome BAC_53G04 | 4 | 2 | 1 | 0 | 0 | 1 | 1 | 9 |
| Y chromosome BAC_57M14 | 4 | 5 | 0 | 1 | 1 | 0 | 1 | 12 |
| Y^h^ chromosome BAC_62H24 | 1 | 0 | 0 | 0 | 0 | 2 | 0 | 3 |
| Y^h^ chromosome BAC_65D15 | 0 | 0 | 1 | 1 | 0 | 0 | 0 | 2 |
| Y^h^ chromosome BAC_71E16 | 1 | 7 | 1 | 0 | 0 | 2 | 4 | 15 |
| Y^h^ chromosome BAC_72J22 | 1 | 2 | 1 | 0 | 0 | 0 | 1 | 5 |
| Y^h^ chromosome BAC_81O12 | 5 | 0 | 0 | 1 | 0 | 1 | 3 | 10 |
| Y^h^ chromosome BAC_PH85B24 | 1 | 1 | 0 | 0 | 0 | 1 | 2 | 5 |
| Y^h^ chromosome BAC_90D06 | 1 | 2 | 1 | 1 | 1 | 1 | 0 | 7 |
| Y chromosome BAC_PH94E22 | 0 | 0 | 0 | 0 | 0 | 0 | 1 | 1 |
| Y^h^ chromosome BAC_PH95B12 | 0 | 0 | 0 | 1 | 1 | 0 | 0 | 2 |
| Y^h^ chromosome BAC_96A24 | 2 | 0 | 1 | 1 | 1 | 1 | 3 | 9 |
| Total number : 15 | 28 | 23 | 11 | 7 | 6 | 14 | 28 | 117 |
