# Supplementary material for: The development of functional mapping by three sex-related loci on the third whorl of different sex types of Carica papaya L
Source: PLoS One. 2018 Mar 22;13(3):e0194605. doi: 10.1371/journal.pone.0194605 (PMC5864051; doi:10.1371/journal.pone.0194605)
Supplement: S2 Table — (DOCX) [file pone.0194605.s017.docx]

| Papaya_BAC_ID | Gene # of AA-seq change | | | Gene # of none  AA-seq change |
| --- | --- | --- | --- | --- |
|  | Exon jump | Intron jump | AA short |  |
| Y^h^ chromosome BAC_49L11 | 0 | 0 | 2 | 0 |
| X chromosome BAC_50J21 | 5 | 1 | 4 | 8 |
| Y^h^ chromosome BAC_50M09 | 1 | 1 | 0 | 0 |
| Y^h^ chromosome BAC_53G04 | 0 | 0 | 1 | 1 |
| Y chromosome BAC_57M14 | 1 | 1 | 1 | 1 |
| Y^h^ chromosome BAC_62H24 | 0 | 0 | 0 | 2 |
| Y^h^ chromosome BAC_65D15 | 1 | 0 | 0 | 0 |
| Y^h^ chromosome BAC_71E16 | 2 | 2 | 1 | 2 |
| Y^h^ chromosome BAC_72J22 | 0 | 0 | 0 | 1 |
| Y^h^ chromosome BAC_81O12 | 1 | 0 | 0 | 4 |
| Y^h^ chromosome BAC_PH85B24 | 1 | 0 | 1 | 1 |
| Y^h^ chromosome BAC_90D06 | 1 | 1 | 1 | 1 |
| Y chromosome BAC_PH94E22 | 0 | 0 | 0 | 1 |
| Y^h^ chromosome BAC_PH95B12 | 0 | 0 | 0 | 2 |
| Y^h^ chromosome BAC_96A24 | 3 | 2 | 3 | 0 |
| Total number : 15 | 16 | 8 | 14 | 24/55 |

Supplementary Table 2. The gene numbers of the different AS-seq types from the transcriptome data of the three sex types refer to the 15 BACs of papaya.
