# Supplementary material for: The development of functional mapping by three sex-related loci on the third whorl of different sex types of Carica papaya L
Source: PLoS One. 2018 Mar 22;13(3):e0194605. doi: 10.1371/journal.pone.0194605 (PMC5864051; doi:10.1371/journal.pone.0194605)
Supplement: S3 Table — type_1: the genes are not expressed in one of the three sex types, and there are differences in their AA-seqs between those of the other two sex types; type_2: the genes are expressed in all three sex types, and there are differences in their AA-seqs; type_3: the genes are expressed in all three sex types, and there is no difference in AA-seqs between two sex types. (DOCX) [file pone.0194605.s018.docx]

| Papaya_BAC_ID | Gene # of different expression seq type | | | Total of gene # |
| --- | --- | --- | --- | --- |
|  | Type 1 | Type 2 | Type 3 |  |
| Y^h^ chromosome BAC_49L11 | 0 | 0 | 2 | 0 |
| X chromosome BAC_50J21 | 4 | 0 | 5 | 9 |
| Y^h^ chromosome BAC_50M09 | 0 | 1 | 0 | 1 |
| Y^h^ chromosome BAC_53G04 | 0 | 1 | 0 | 1 |
| Y chromosome BAC_57M14 | 1 | 1 | 0 | 2 |
| Y^h^ chromosome BAC_62H24 | 0 | 0 | 0 | 0 |
| Y^h^ chromosome BAC_65D15 | 1 | 0 | 0 | 1 |
| Y^h^ chromosome BAC_71E16 | 2 | 1 | 1 | 4 |
| Y^h^ chromosome BAC_72J22 | 0 | 0 | 0 | 0 |
| Y^h^ chromosome BAC_81O12 | 0 | 1 | 0 | 1 |
| Y^h^ chromosome BAC_PH85B24 | 0 | 1 | 1 | 2 |
| Y^h^ chromosome BAC_90D06 | 2 | 0 | 0 | 2 |
| Y chromosome BAC_PH94E22 | 0 | 0 | 0 | 0 |
| Y^h^ chromosome BAC_PH95B12 | 0 | 0 | 0 | 0 |
| Y^h^ chromosome BAC_96A24 | 3 | 1 | 2 | 6 |
| Total number : 15 | 13/31 | 7/31 | 11/31 | 31 |

Supplementary Table 3. The gene numbers of the different sex groupings and AS-seq types from the transcriptome data of the three sex types refer to the 15 BACs of papaya.

type_1: the genes are not expressed in one of the three sex types, and there are differences in their AA-seqs between those of the other two sex types; type_2: the genes are expressed in all three sex types, and there are differences in their AA-seqs; type_3: the genes are expressed in all three sex types, and there is no difference in AA-seqs between two sex types.
