# Supplementary material for: The development of functional mapping by three sex-related loci on the third whorl of different sex types of Carica papaya L
Source: PLoS One. 2018 Mar 22;13(3):e0194605. doi: 10.1371/journal.pone.0194605 (PMC5864051; doi:10.1371/journal.pone.0194605)
Supplement: S4 Table — (DOCX) [file pone.0194605.s019.docx]

| Primer name | Forward | Reverse | Genotype | | | Position (base-pair) |
| --- | --- | --- | --- | --- | --- | --- |
|  |  |  | F | M | H |  |
| CpSVPL_HRM_88 | AATAAAATGCATAATTACATAGCACAG | AGAAAGGTCAGTTTACCACATAA | X | G | T | 8487 |
| CpSVPL_RFLP_Nde I | CTGCAAAGTAATGTTCTTTAAGCaTAT | AATAAAATGCATAATTACATAGCACAG | X | G | T | 8487 |
| CpSERK_HRM_30704 | TTCATTGAGATGCGTATTG | GCTCATCACAAGTAAAGTC | A | G | G | 122536 |
| CpSERK_HRM_34072 | CTCCTCCACCATTTGTTC | GCTATTGATGTGTTGCTAC | T | T | C | 119737 |
|  |  |  | A | C | C | 119742 |
| CpSERK_RFLP_Spe I | TGTTGCTACAAAATTCAATATAcTAG | TTAGGTTATTCCGTGACTATC | T | T | C | 119737 |
| CpSERK_HRM_34760 | TTTTGGGTAGAGGAGGATTT | AGCTACTAGTGTACCATCAG | C | T | T | 119048 |
| CpSERK_HRM_34787 | TAGCTGATGGTACACTAGTA | TCTACTTCTGTCTGAAACTG | G | A | A | 119022 |
| CpCAF1AL_HRM_01 | CTGTTAGCAGGTAAAGGC | TGTATCAGATGGAGGCAT | G | A | G | 144693 |
| CpCAF1AL_HRM_02 | TGCCTCCATCTGATACAAGTA | AACTAGTCTGCCCCTGTG | C | T | C | 144403 |
| CpCAF1AL_HRM_04 | GAAGATGAATGTGCAGATGG | AGGATGTCCATTAGCTCAAA | AGA | - | AGA | 359472 |

Supplementary Table 4. Genotyping of three sex types, primer name and primer sequence for SNP-HRM assay on the sex-related genes of papaya.
