# Supplementary material for: The development of functional mapping by three sex-related loci on the third whorl of different sex types of Carica papaya L
Source: PLoS One. 2018 Mar 22;13(3):e0194605. doi: 10.1371/journal.pone.0194605 (PMC5864051; doi:10.1371/journal.pone.0194605)
Supplement: S5 Table — (DOCX) [file pone.0194605.s020.docx]

Supplementary Table 5. The primer name and sequence for RT-PCR and qPCR assays of the sex-related genes of papaya.

| Gene name | |  | | Test | | Primer name | | Forward | | Primer name | | Reverse | |
| --- | --- | --- | --- | --- | --- | --- | --- | --- | --- | --- | --- | --- | --- |
| CpSVPL | | RT-PCR | | Intron 1 | | CpSVPL_JspF | | CATATGTTGCACGAAAAGAAC | | CpSVPL_J2cR | | CCCTCATTTGCTTCAAGTG | |
|  | |  | | Intron 2 | | CpSVPL_J1F | | TGAACTAAAGCTAGTTGAGAATG | | CpSVPL_I2R | | CCCTCATTTGCTTCAAGTG | |
|  | |  | | Intron 3 | | CpSVPL_J2F | | CACTTGAAGCAAATGAGGG | | CpSVPL_I3R | | CCTTCCTTTGAAGATCACTG | |
|  | |  | | Intron 4 | | CpSVPL_J3F | | GTTATAGAGAGAAAGGGTCAAAAT | | CpSVPL_I4R | | TGTTGCCTCAGTTTCTCAT | |
|  | |  | | Intron 5 | | CpSVPL_J4F | | TCAAAGGAAGGGAATACAATTG | | CpSVPL_I5R | | ATTGGTTACAGACTCTGATGA | |
|  | |  | | Intron 6 | | CpSVPL_J5F | | AGGCAACAATTGTTGCAG | | CpSVPL_I6R | | GAATAGGGTAACCCAAGCT | |
|  | | qPCR | | Junction 1 | | CpSVPL_J1F | | TGAACTAAAGCTAGTTGAGAATG | | CpSVPL_J1R | | TTCAAGTGATGGCTTCTCT | |
|  | |  | | Junction 2 | | CpSVPL_J2F | | CACTTGAAGCAAATGAGGG | | CpSVPL_J2R | | CTATAACACGGCGCAATC | |
|  | |  | | Junction 3 | | CpSVPL_J3F | | GTTATAGAGAGAAAGGGTCAAAAT | | CpSVPL_J3R | | CCTTCCTTTGAAGATCACTG | |
|  | |  | | Junction 4 | | CpSVPL_J4F | | TCAAAGGAAGGGAATACAATTG | | CpSVPL_J4R | | TGTTGCCTCAGTTTCTCAT | |
|  | |  | | Junction 5 | | CpSVPL_J5F | | AGGCAACAATTGTTGCAG | | CpSVPL_J5R | | ATTGGTTACAGACTCTGATGA | |
|  | |  | | Junction 6 | | CpSVPL_J6F | | GAAGAGGGCCAGTCATC | | CpSVPL_J6R | | GAATAGGGTAACCCAAGCT | |
| CpSERK | | RT-PCR | | Intron 1 | | CpSERK_JspF | | TTCCTGCTGCATCCATTA | | CpSERK_J2cR | | CCTAGATCAACTCGTATGACA | |
|  | |  | | Intron 2 | | CpSERK_J1F | | ACATGGAAGGTGATGCAC | | CpSERK_J2R | | CCAAGCTGTGGAACCA | |
|  | |  | | Intron 3 | | CpSERK_J2F | | TGTCATACGAGTTGATCTAGG | | CpSERK_J3R | | GATGGGACCACTGAAACT | |
|  | |  | | Intron 4 | | CpSERK_J3F | | GCAGTATTTGGAACTCTACAG | | CpSERK_J4R | | TTGGAGGGATGAGATATTAGTC | |
|  | |  | | Intron 5 | | CpSERK_J4F | | CGCTTTCTTCGGCTTAAC | | CpSERK_J5R | | CAGTGAAAAGGAGCCATTATC | |
|  | |  | | Intron 6 | | CpSERK_J5F | | CTCCAAGTACTGGATCTATCAA | | CpSERK_J6R | | AAGAAATTGGAGGTGGG | |
|  | |  | | Intron 7 | | CpSERK_J6F | | CATTAGTTTTGCCAACAACT | | CpSERK_J7R | | GGTTTCCTCCTACGCC | |
|  | |  | | Intron 8 | | CpSERK_J7F | | CCAGGTGGGAATAGTGC | | CpSERK_J8R | | TAAACCTTACCAAATCCTCCT | |
|  | |  | | Intron 9 | | CpSERK_J8F | | TGATGTACCTGCTGAAGAG | | CpSERK_J9R | | ATCATGCAAGTAAGAAAGACC | |
|  | |  | | Intron 10 | | CpSERK_J9F | | CATGTTTAAGAGAACGTCCTC | | CpSERK_J10R | | TGGATTAATTGCTCTACCTCAG | |
|  | | qPCR | | Junction 1 | | CpSERK_J1F | | ACATGGAAGGTGATGCAC | | CpSERK_J1R | | CTCGTATGACACTATTATCATTGT | |
|  | |  | | Junction 2 | | CpSERK_J2F | | TGTCATACGAGTTGATCTAGG | | CpSERK_J2R | | CCAAGCTGTGGAACCA | |
|  | |  | | Junction 3 | | CpSERK_J3F | | GCAGTATTTGGAACTCTACAG | | CpSERK_J3R | | GATGGGACCACTGAAACT | |
|  | |  | | Junction 4 | | CpSERK_J4F | | CGCTTTCTTCGGCTTAAC | | CpSERK_J4R | | TTGGAGGGATGAGATATTAGTC | |
|  | |  | | Junction 5 | | CpSERK_J5F | | CTCCAAGTACTGGATCTATCAA | | CpSERK_J5R | | CAGTGAAAAGGAGCCATTATC | |
|  | |  | | Junction 6 | | CpSERK_J6F | | CATTAGTTTTGCCAACAACT | | CpSERK_J6R | | AAGAAATTGGAGGTGGG | |
|  | |  | | Junction 7 | | CpSERK_J7F | | CCAGGTGGGAATAGTGC | | CpSERK_J7R | | GGTTTCCTCCTACGCC | |
|  | |  | | Junction 8 | | CpSERK_J8F | | TGATGTACCTGCTGAAGAG | | CpSERK_J8R | | TAAACCTTACCAAATCCTCCT | |
|  | |  | | Junction 9 | | CpSERK_J9F | | CATGTTTAAGAGAACGTCCTC | | CpSERK_J9R | | ATCATGCAAGTAAGAAAGACC | |
|  | |  | | Junction 10 | | CpSERK_J10F | | ACTTGATTGGGTGAAAGGG | | CpSERK_J10R | | TGGATTAATTGCTCTACCTCAG | |
| CpCAF1AL | | RT-PCR | | Intron 1 | | CpCAF1AL_JspF | | GACGATACTGAATCTTGCCTCT | | CpCAF1AL_J2cR | | TTATCATTGCGGAGACAGCA | |
|  | |  | | Intron 2 | | CpCAF1AL_J1F | | GTGCTGGGAGACAACTGA | | CpCAF1AL_J2R | | TCTGGTTCACTTAAGACTTTACGA | |
|  | |  | | Intron 3 | | CpCAF1AL_J2F | | TGCTGTCTCCGCAATGATAA | | CpCAF1AL_J3R | | TTTCACTGTTTCACGGTCC | |
|  | |  | | Intron 4 | | CpCAF1AL_J3F | | GTGCAGAAGTGAGTGAGAAG | | CpCAF1AL_J4R | | TCAGCATGGCTTGATGAA | |
|  | |  | | Intron 5 | | CpCAF1AL_J4F | | GGCAAACAGAAAAAGAGCA | | CpCAF1AL_J5R | | CCAATCCATCGATGTTAAACT | |
|  | |  | | Intron 6 | | CpCAF1AL_J5F | | TTCGCAAGTTGCACTTG | | CpCAF1AL_J6R | | ATAATCCAAATCCGGGTCCTT | |
|  | |  | | Intron 7 | | CpCAF1AL_J6F | | GGCCTAAGAAAAGTCAAGTTGT | | CpCAF1AL_J7R | | CTTTATCACAATCTGAGAGGCTTT | |
|  | |  | | Intron 8 | | CpCAF1AL_J7F | | AGAATGGGAAGAGGAAGAACC | | CpCAF1AL_J8R | | AAGTCTTGCTTACAATTAGGTGAA | |
|  | |  | | Intron 9 | | CpCAF1AL_J8F | | CAGAAAATGAGGGAGTGCC | | CpCAF1AL_J9R | | ACCTTGGAATATCAGGGAACT | |
|  | |  | | Intron 10 | | CpCAF1AL_J9F | | AATTTGGTTGAAATGGTCTCTCTA | | CpCAF1AL_J10R | | TGAAGTTGAAAAACCAAGTTTGAC | |
|  | |  | | Intron 11 | | CpCAF1AL_J10F | | GCTGGCAGGTCAAGAAAG | | CpCAF1AL_J11R | | AGCATTACCTGGTTTTAGAGACT | |
|  | | qPCR | | Junction 1 | | CpCAF1AL_J1F | | GTGCTGGGAGACAACTGA | | CpCAF1AL_J1R | | GTACTCCTCGTGCAGATTTTG | |
|  | |  | | Junction 2 | | CpCAF1AL_J2F | | TGCTGTCTCCGCAATGATAA | | CpCAF1AL_J2R | | TCTGGTTCACTTAAGACTTTACGA | |
|  | |  | | Junction 3 | | CpCAF1AL_J3F | | GTGCAGAAGTGAGTGAGAAG | | CpCAF1AL_J3R | | TTTCACTGTTTCACGGTCC | |
|  | |  | | Junction 4 | | CpCAF1AL_J4F | | GGCAAACAGAAAAAGAGCA | | CpCAF1AL_J4R | | TCAGCATGGCTTGATGAA | |
|  | |  | | Junction 5 | | CpCAF1AL_J5F | | TTCGCAAGTTGCACTTG | | CpCAF1AL_J5R | | CCAATCCATCGATGTTAAACT | |
|  | |  | | Junction 6 | | CpCAF1AL_J6F | | GGCCTAAGAAAAGTCAAGTTGT | | CpCAF1AL_J6R | | ATAATCCAAATCCGGGTCCTT | |
|  | |  | | Junction 7 | | CpCAF1AL_J7F | | AGAATGGGAAGAGGAAGAACC | | CpCAF1AL_J7R | | CTTTATCACAATCTGAGAGGCTTT | |
|  | |  | | Junction 8 | | CpCAF1AL_J8F | | CAGAAAATGAGGGAGTGCC | | CpCAF1AL_J8R | | AAGTCTTGCTTACAATTAGGTGAA | |
|  | |  | | Junction 9 | | CpCAF1AL_J9F | | AATTTGGTTGAAATGGTCTCTCTA | | CpCAF1AL_J9R | | ACCTTGGAATATCAGGGAACT | |
|  | |  | | Junction 10 | | CpCAF1AL_J10F | | GCTGGCAGGTCAAGAAAG | | CpCAF1AL_J10R | | TGAAGTTGAAAAACCAAGTTTGAC | |
|  | |  | | Junction 11 | | CpCAF1AL_J11F | | TTCAACTTCACCAAGTAAAGGC | | CpCAF1AL_J11R | | AGCATTACCTGGTTTTAGAGACT | |
