# Supplementary material for: The development of functional mapping by three sex-related loci on the third whorl of different sex types of Carica papaya L
Source: PLoS One. 2018 Mar 22;13(3):e0194605. doi: 10.1371/journal.pone.0194605 (PMC5864051; doi:10.1371/journal.pone.0194605)
Supplement: S6 Table — 28 days before flowering: different sex types at 28 days before flowering; 0 days before flowering: different sex types at 0 days before flowering; ND: not determined; -: no difference; x: no primer design; F: different female; M: different male; H: different hermaphrodite; HM3: different female degradation hermaphrodite; 4: fourth whorl of flower; 3: third whorl of flower. (DOCX) [file pone.0194605.s021.docx]

Supplementary Table 6. The summary of the RT-PCR and Q-PCR analysis results of the flowers of different sex types in the 4^th^ and 3^rd^ whorls of flowers.

| Test | | Sex relative expression | | | | | | Hermaphrodite forms relative expression | | | | | |
| --- | --- | --- | --- | --- | --- | --- | --- | --- | --- | --- | --- | --- | --- |
|  |  | CpSVPL | | CpSERK | | CpCAF1AL | | CpSVPL | | CpSERK | | CpCAF1AL | |
|  |  | 28 | 0 | 28 | 0 | 28 | 0 | 28 | 0 | 28 | 0 | 28 | 0 |
| RT-PCR intron test | Intron 1 | _ | _ | _ | _ | _ | _ | _ | _ | _ | _ | _ | _ |
|  | Intron 2 | _ | _ | _ | _ | _ | _ | _ | _ | _ | _ | _ | _ |
|  | Intron 3 | _ | _ | _ | _ | _ | _ | _ | _ | _ | _ | _ | _ |
|  | Intron 4 | F | _ | _ | _ | _ | _ | _ | _ | _ | _ | _ | _ |
|  | Intron 5 | _ | _ | _ | _ | _ | _ | _ | _ | _ | _ | _ | _ |
|  | Intron 5-6 | ND | ND | _ | 3,4 | ND | ND | ND | ND | _ | H(3) | ND | ND |
|  | Intron 6 | F | F(4) | _ | _ | _ | _ | _ | _ | _ | _ | _ | _ |
|  | Intron 6-7 | x | x | _ | 3,4 | ND | ND | x | x | _ | H(3) | ND | ND |
|  | Intron 7 | x | x | _ | _ | _ | _ | x | x | _ | _ | _ | _ |
|  | Intron 8 | x | x | _ | _ | _ | _ | x | x | _ | _ | _ | _ |
|  | Intron 9 | x | x | _ | _ | _ | _ | x | x | _ | _ | _ | _ |
|  | Intron 10 | x | x | _ | _ | _ | _ | x | x | _ | _ | _ | _ |
|  | Intron 11 | x | x | x | x | _ | 3,4 | x | x | x | x | _ | H(3) |
| Q-PCR junction test | Junction 1 | _ | _ | _ | _ | _ | _ | _ | _ | _ | _ | _ | _ |
|  | Junction 2 | _ | _ | _ | _ | _ | _ | _ | _ | _ | _ | _ | _ |
|  | Junction 3 | _ | _ | _ | _ | _ | _ | _ | _ | _ | _ | _ | _ |
|  | Junction 4 | _ | _ | _ | _ | _ | 3,4 | _ | _ | _ | _ | _ | 3,4 |
|  | Junction 5 | _ | _ | _ | _ | _ | _ | _ | _ | _ | _ | _ | _ |
|  | Junction 6 | _ | M(3) | _ | _ | _ | _ | _ | HM(3) | _ | _ | _ | _ |
|  | Junction 7 | x | x | _ | _ | _ | _ | x | x | _ | _ | _ | _ |
|  | Junction 8 | x | x | _ | _ | _ | _ | x | x | _ | _ | _ | _ |
|  | Junction 9 | x | x | _ | _ | _ | _ | x | x | _ | _ | _ | _ |
|  | Junction 10 | x | x | _ | _ | _ | _ | x | x | _ | _ | _ | _ |
|  | Junction 11 | x | x | x | x | _ | _ | x | x | x | x | _ | _ |

28: Different sex-type of 28 days before flowering buds; 0: Different sex-type of 0 days before flowering buds; ND: not determination; -: no difference; x: no primer design; F: differentiating female; M: differentiating male; H: differentiating hermaphrodites; HM3: differentiating female degradation hermaphrodite; 4: 4^th^ whorl of flower; 3: 3^th^ whorl of flower.
