# Supplementary material for: The development of functional mapping by three sex-related loci on the third whorl of different sex types of Carica papaya L
Source: PLoS One. 2018 Mar 22;13(3):e0194605. doi: 10.1371/journal.pone.0194605 (PMC5864051; doi:10.1371/journal.pone.0194605)
Supplement: S7 Table — Gray area: lowest expression junction of the CpSVPL gene. (DOCX) [file pone.0194605.s022.docx]

Supplementary Table 7. The average expression of each junction of the *CpSVPL* gene based on the results of the qPCR assay using three sample repeats of twelve samples.

| qPCR test | Junction 1 | Junction 2 | Junction 3 | Junction 4 | Junction 5 | Junction 6 |
| --- | --- | --- | --- | --- | --- | --- |
| 1^st^ | 54.53±91.19 | 0.03±0.04 | 0.02±0.036 | 0.03±0.04 | 0.0001±0.0001 | 0.0002±0.0004 |
| 2^nd^ | 30.71±40.81 | 0.02±0.02 | 0.16±0.40 | 0.02±0.01 | 0.0001±0.0001 | 0.0007±0.001 |
| 3^th^ | 134.35±132.99 | 0.05±0.08 | 0.14±0.24 | 0.20±0.62 | 0.0005±0.0003 | 0.0009±0.0009 |

Gray area: lowest expression junction of *CpSVPL* gene.
