# Supplementary material for: The development of functional mapping by three sex-related loci on the third whorl of different sex types of Carica papaya L
Source: PLoS One. 2018 Mar 22;13(3):e0194605. doi: 10.1371/journal.pone.0194605 (PMC5864051; doi:10.1371/journal.pone.0194605)
Supplement: S8 Table — Gray area: lowest expression junction of the CpSERK gene. (DOCX) [file pone.0194605.s023.docx]

Supplementary Data Table 8. The average expression of each junction of the *CpSERK* gene based on the results of the qPCR assay using three sample repeats of twelve samples.

| qPCR test | Junction 1 | Junction 2 | Junction 3 | Junction 4 | Junction 5 | Junction 6 | Junction 7 | Junction 8 | Junction 9 | Junction 10 |
| --- | --- | --- | --- | --- | --- | --- | --- | --- | --- | --- |
| 1^st^ | 0.05±0.03 | 0.14±0.08 | 0.82±0.59 | 0.64±1.09 | 0.08±0.04 | 0.23±0.17 | 0.01±0.004 | 0.05±0.01 | 0.09±0.07 | 0.01±0.004 |
| 2^nd^ | 0.18±0.27 | 1.13±1.63 | 3.90±4.33 | 0.14±0.13 | 2.74±5.11 | 2.88±4.94 | 0.27±0.70 | 0.50±0.75 | 1.79±2.65 | 0.26±0.61 |
| 3^th^ | 0.20±0.20 | 0.63±1.13 | 0.47±0.42 | 0.08±0.06 | 1.15±2.90 | 0.25±0.32 | 0.02±0.008 | 0.14±0.29 | 7.81±7.93 | 0.01±0.005 |

Gray area: lowest expression junction of *CpSERK* gene.
