# Supplementary material for: The development of functional mapping by three sex-related loci on the third whorl of different sex types of Carica papaya L
Source: PLoS One. 2018 Mar 22;13(3):e0194605. doi: 10.1371/journal.pone.0194605 (PMC5864051; doi:10.1371/journal.pone.0194605)
Supplement: S9 Table — Gray area: lowest expression junction of the CpCAF1AL gene. (DOCX) [file pone.0194605.s024.docx]

Supplementary Data Table 9. The average expression of each junction of the *CpCAF1AL* gene based on the results of the qPCR assay using three sample repeats of twelve samples.

| qPCR test | Junction 1 | Junction 2 | Junction 3 | Junction 4 | Junction 5 | Junction 6 | Junction 7 | Junction 8 | Junction 9 | Junction 10 | Junction 11 |
| --- | --- | --- | --- | --- | --- | --- | --- | --- | --- | --- | --- |
| 1^st^ | 0.006±0.009 | 0.0007±0.0004 | 0.002±0.002 | 0.0003±0.0002 | 0.00006±0.00006 | 0.02±0.011 | 9.96±15.89 | 0.31±0.33 | 0.19±0.18 | 0.10±0.064 | 0.01±0.01 |
| 2^nd^ | 0.002±0.002 | 0.0009±0.0009 | 0.07±0.19 | 0.0003±0.0003 | 0.0004±0.0005 | 0.32±0.30 | 4.93±6.41 | 1.54±1.59 | 0.22±0.22 | 0.004±0.004 | 0.003±0.009 |
| 3^th^ | 0.001±0.0006 | 0.001±0.0005 | 0.02±0.04 | 0.0003±0.0002 | 0.0002±0.0002 | 0.65±1.54 | 0.02±0.03 | 0.015±0.045 | 0.001±0.001 | 0.01±0.05 | 0.0013±0.002 |

Gray area: lowest expression junction of *CpCAF1AL* gene.
